# Furan-Containing Chiral Spiro-Fused Polycyclic Aromatic Compounds: Synthesis and Photophysical Properties

**DOI:** 10.3390/molecules27165103

**Published:** 2022-08-11

**Authors:** Koji Nakano, Ko Takase, Keiichi Noguchi

**Affiliations:** 1 Department of Applied Chemistry, Graduate School of Engineering, Tokyo University of Agriculture and Technology, 2-24-16 Naka-cho, Koganei, Tokyo 184-8588, Japan; 2 Instrumentation Analysis Center, Tokyo University of Agriculture and Technology, 2-24-16 Naka-cho, Koganei, Tokyo 184-8588, Japan

**Keywords:** spiro π-conjugated compound, chiral compound, thiophene, furan, pyrrole, nucleophilic aromatic substitution, circular dichroism, circularly polarized luminescence

## Abstract

Spiro-fused polycyclic aromatic compounds (PACs) have received growing interest as rigid chiral scaffolds. However, furan-containing spiro-fused PACs have been quite limited. Here, we design spiro[indeno[1,2-*b*][1]benzofuran-10,10′-indeno[1,2-*b*][1]benzothiophene] as a new family of spiro-fused PACs that contains a furan unit. The compound was successfully synthesized in enantiopure form and also transformed to its *S*,*S*-dioxide derivative and the pyrrole-containing analog via aromatic metamorphosis. The absorption and emission properties of the obtained furan-containing chiral spiro-fused PACs are apparently different from those of their thiophene analogs that have been reported, owing to the increased electron-richness of furan compared to thiophene. All of the furan-containing chiral spiro-fused PACs were found to be circularly polarized luminescent materials.

## 1. Introduction

Spiro-fused polycyclic aromatic compounds (PACs), in which two planarized biaryls are connected perpendicularly by a tetragonal spiro atom, have been intensively studied in the last few decades, owing to their unique three-dimensional structure, high thermal stability, superior processability, and promising photophysical and electronic properties [1,2,3]. These intriguing characteristics offer advantages in (opto)electronic applications, such as organic field effect transistors [4,5], organic light emitting diodes [6,7,8], and organic solar cells [9,10,11,12]. The prototypical motif of spiro-fused PACs is 9,9′-spirobi[fluorene] (SBF). A variety of SBF-based compounds with functional substituent(s) and/or a π-extended structure have been developed for the purpose of tuning the photophysical and electronic properties [1,2]. Incorporation of heterocycle unit(s) into π-conjugated systems or a heteroatom as a spiro atom has also been a promising strategy to achieve the desired functions of spiro-fused PACs [3,13,14,15,16].

**Figure 1:**
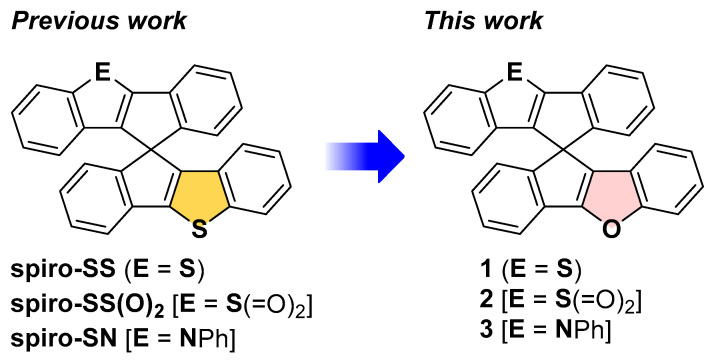
Molecular structures of previously reported chiral spiro-fused PACs and **1**–**3** in this study.

When two dissymmetric biaryls are linked by a spiro atom, the resulting spiro compound is chiral. Two different dissymmetric biaryls give a *C*_1_-symmetric spiro compound, while two identical dissymmetric biaryls afford a *C*_2_-symmetric one. Although chiral spiro-fused PACs have attracted less attention until recently, there have been several reports on their applications to molecular recognition [17,18], diastereoselective self-assembly [19,20], and asymmetric catalysts [21,22]. In 2016, Kuninobu, Takai, and co-workers reported the first example of a chiral spiro-fused PAC [23] that exhibited circularly polarized luminescence (CPL), which has received significant interest, owing to its potential applications [24,25,26,27,28]. This report has triggered many studies on spiro-fused PACs that exhibit CPL [29,30,31,32,33,34,35,36,37]. We have also developed a series of chiral spiro-fused PACs, such as **spiro-SS**, **-SS(O)_2_**, and **-SN** (Figure 1) [16,38,39,40,41,42]. These spiro-fused PACs exhibit CPL and their optical properties were found to depend on the substituents and the incorporated heterocycle units. The recent studies clearly demonstrate the excellent potential of spiro-fused PACs as chiral materials, and further investigations are still required for elucidating the structure–property relationship, and thus developing spiro-fused PACs with tailored properties.

In the course of our studies, we have focused on incorporating a furan unit in a chiral spiro-fused PAC skeleton. Furan, the oxygen analog of thiophene, has reduced aromaticity and is more electron-rich compared to thiophene [43]. Therefore, the electronic perturbation of a furan unit to a π-conjugated system would be different from that of a thiophene unit, inducing unique electronic and photophysical properties. To date, a wide range of furan-containing π-conjugated compounds have been reported as organic functional materials [44,45,46,47,48]. However, the synthetic attempt of spiro-fused PACs containing furan unit(s) have been quite limited. In 2010, Ohe and co-workers reported the first examples of furan-containing spiro-fused PACs [49,50]. These compounds were found to be highly emissive. More recently, the chiral spiro-fused PAC with a furan unit has been reported by Nakamura and co-workers, demonstrating CPL properties [35]. Herein, we report the synthesis and photophysical properties of furan-containing chiral spiro-fused PACs **1**–**3**, which are oxygen analogs of **spiro-SS**, **-SS(O)_2_**, and **-SN** (Figure 1). The *S*,*S*-dioxide derivative **2** and the pyrrole-containing compound **3** can be synthesized from **1** through aromatic metamorphosis. Incorporation of a furan unit in place of a thiophene unit was found to have great impact on photophysical properties.

## 2. Results

**Scheme 1:**
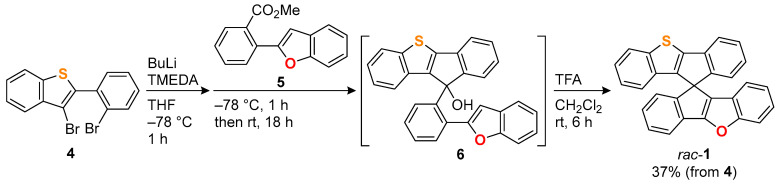
Synthesis of *rac*-**1**.

**Figure 2:**
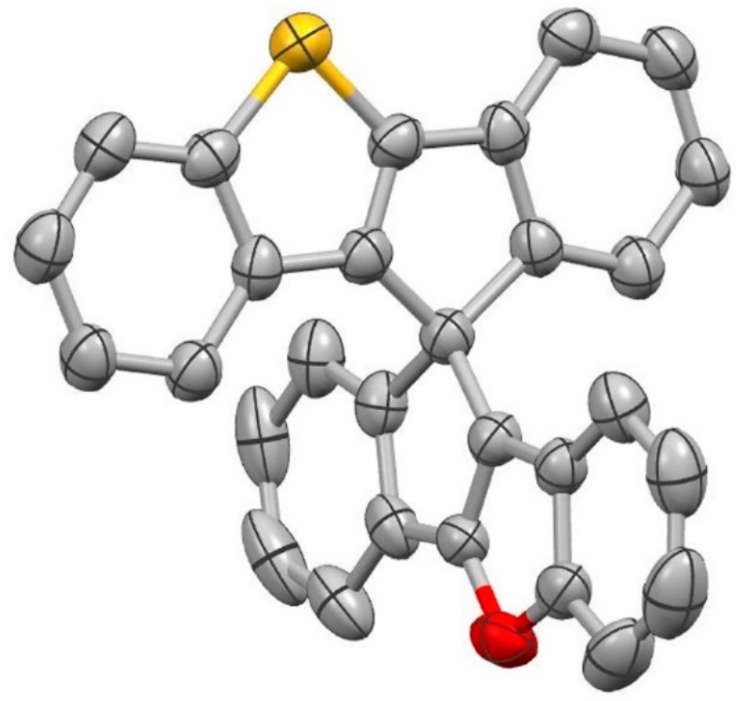
ORTEP drawing of *rac*-**1** with thermal ellipsoids at 50% probability. All hydrogen atoms are omitted for clarity.

Our first attempt to prepare racemic **1** (*rac*-**1**) is illustrated in Scheme 1. 3-Bromo-2-(bromophenyl)-1-benzothiophene (**4**) was dilithiated with BuLi, and then treated with the ester **5**. The subsequent Friedel–Crafts cyclization of the resulting tertiary alcohol **6** successfully gave the desired compound *rac*-**1** (37% yield from **4**). The structure was confirmed by X-ray crystallographic analysis (Figure 2). Both enantiomers were found to be contained in the unit cell. The two spiro-linked π-conjugated planes are almost completely perpendicular (87.3°). Screening of optical resolution conditions with HPLC on a chiral stationary phase (chiral HPLC) showed that baseline separation of enantiomers cannot be achieved.

**Scheme 2:**
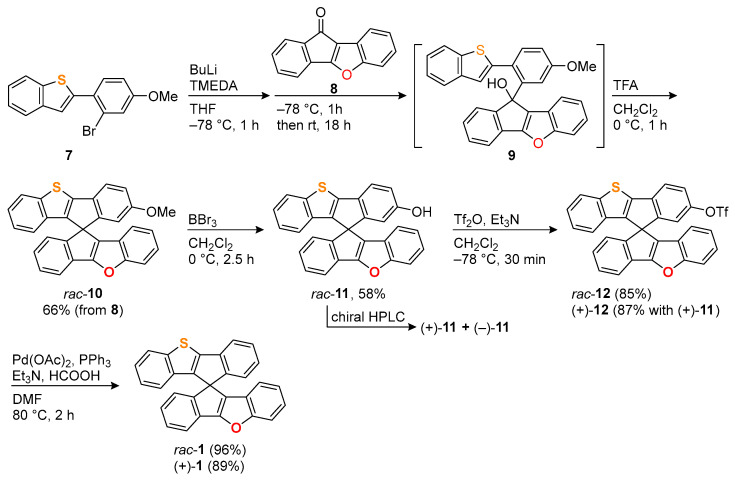
Synthesis of the hydroxy-substituted spiro compound **11** and its transformation to **1**.

In order to prepare each enantiomer of **1**, we designed the hydroxy-substituted spiro compound **11,** which would be converted into **1** via a two-step reaction (Scheme 2). Previously, Lützen and co-workers have reported the efficient optical resolution of 9,9′-spirobi[fluorene]-2,2′-diol and its derivatives with chiral HPLC [51]. We have also demonstrated that the dihydroxylated derivative of **spiro-SS** can be separated into enantiomers with chiral HPLC more efficiently than its parent compound **spiro-SS** [39]. Furthermore, Nakamura and co-workers reported the optical resolution of the furan-containing chiral spiro-fused PAC with one hydroxy group by using chiral HPLC [35]. Accordingly, we envisaged that incorporation of a hydroxy group on **1** would make an efficient optical resolution with chiral HPLC possible.

The synthesis of the hydroxy-substituted compound **11** and its transformation to **1** are illustrated in Scheme 2. First, 2-(2-bromo-4-methoxyphenyl)-1-benzothiophene (**7**) was lithiated with BuLi, and then treated with 10*H*-indeno[1,2-*b*][1]benzofuran-10-one (**8**). The resulting tertiary alcohol **9** was converted into the methoxy-substituted spiro compound *rac*-**10** via acid-promoted Friedel–Crafts cyclization (66% yield from **8**). Finally, demethylation of *rac*-**10** with BBr_3_ gave the hydroxy-substituted compound **11** (58% yield) in a racemic form. As expected, the optical resolution of *rac*-**11** was achieved by HPLC with a CHIRALPAK^®^ IA column with hexane/CHCl_3_ (50/50) as an eluent (Figure S18). In addition, the hydroxy group of **11** was found to be cleaved off via the transformation to the triflate **12** (85% yield with *rac*-**11**; 87% yield with (+)-**11**) and the following palladium-catalyzed reduction with formic acid (96% yield with *rac*-**12**; 89% yield with (+)-**12**) [39]. This two-step reaction should not affect the chiral center. Therefore, the transformation of enantiopure **11** can give the corresponding enantiomer of **1** without racemization.

**Scheme 3:**
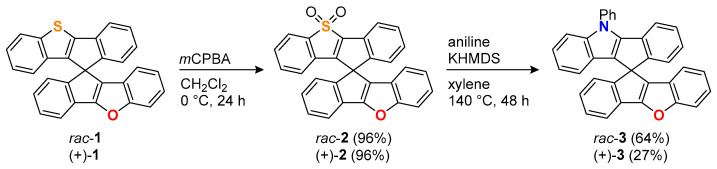
Synthesis of the *S*,*S*-dioxide derivative **2** and the pyrrole-containing compound **3**.

A benzothiophene skeleton can be transformed to an indole one by aromatic metamorphosis, which includes oxidation of a thiophene unit and the inter/intra molecular S_N_Ar reaction of the resulting *S*,*S*-dioxide unit with a primary amine [52]. Recently, we applied this transformation to **spiro-SS** [40]. The resulting spiro-fused compounds with one [**spiro-SS(O)_2_**] or two *S*,*S*-dioxide units or with one (**spiro-SN**) or two pyrrole units showed photophysical properties that were quite different from the parent compound **spiro-SS**. In this context, we investigated the transformation of **1** to the *S*,*S*-dioxide derivative **2** and the pyrrole-containing compound **3** (Scheme 3). The oxidation of *rac*-**1** was readily achieved by using an excess amount of 3-chloroperbenzoic acid (*m*CPBA) as an oxidant, affording *rac*-**2** in high yield (96%). Furthermore, the reaction of *rac*-**2** with aniline in the presence of KHMDS (potassium hexamethyldisilazide) successfully gave *rac*-**3** in a moderate yield (64%). Enantiomers of **2** and **3** were also prepared from enantiopure **1**. The oxidation and the inter/intra molecular S_N_Ar reaction should not affect the chiral center. Therefore, these transformations could proceed without racemization to afford enantiopure **2** and **3**.

**Figure 3:**
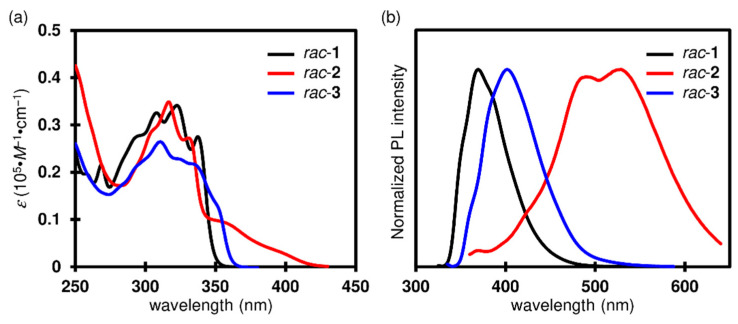
(**a**) UV–vis absorption and (**b**) PL spectra of *rac*-**1–3** in CH_2_Cl_2_.

The UV–vis absorption and photoluminescence (PL) spectra of spiro-fused PACs **1**–**3** are shown in Figure 3. The photophysical data are summarized in Table 1, together with those of the previously reported **spiro-SS**, **spiro-SS(O)_2_**, and **spiro-SN** for comparison. We also performed theoretical calculations by density functional theory (DFT) and time-dependent (TD) DFT methods at the B3LYP/6-31G(d) level of theory to understand the experimental photophysical properties.

Compound *rac*-**1** gave a well-resolved absorption spectrum, with the longest absorption maximum (λ_abs_) at 337 nm (Figure 3a). In contrast, the absorption spectrum of the *S*,*S*-dioxide derivative *rac*-**2** exhibited well-resolved absorption bands in the <340 nm range and broad absorption bands in the longer wavelength range. Such a difference in the absorption properties between *rac*-**1** and *rac*-**2** is the same as that observed for **spiro-SS** and **spiro-SS(O)_2_** [38]. By analogy with the discussion on **spiro-SS** and **spiro-SS(O)_2_** in our previous report, the well-resolved absorption bands and the broader absorption bands were derived from the indeno[1,2-*b*][1]benzofuran subunit and the indeno[1,2-*b*][1]benzothiophene *S*,*S*-dioxide subunit, respectively. Each of these two subunits works as an almost-independent chromophore, since their perpendicular arrangement through a spiro carbon atom allows a limited orbital interaction between them in the ground state. The absorption spectrum of *rac*-**3** is slightly broader and red-shifted in comparison to that of *rac*-**1**, exhibiting a shoulder peak at 353 nm. The absorption spectra of *rac*-**1**–**3** are almost independent of solvent polarity (Figure S19a–c). The TD-DFT calculations demonstrated that the spiro-fused PACs *rac*-**1**–**3** exhibit the calculated longest absorption bands at 352 nm, 428 nm, and 361 nm, respectively, all of which are assigned to the transitions dominated by the HOMO→LUMO transition (Table S5). The obtained calculation results are qualitatively coincident with their experimental absorption spectra. The absorption spectra of *rac*-**1**–**3** are very similar to those of their thiophene analogs **spiro-SS**, **-SS(O)_2_**, and **-SN**, respectively [38,40]. Therefore, the replacement of a thiophene unit with a furan unit was found to have little impact on absorption properties.

**Figure 4:**
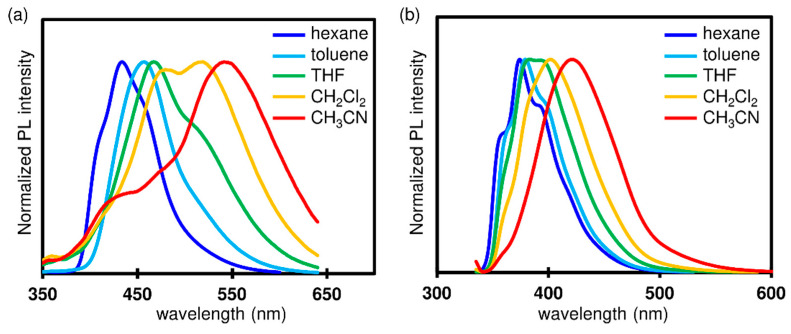
Solvent effect on PL spectra of (**a**) *rac*-**2** and (**b**) *rac*-**3**.

**Figure 5:**
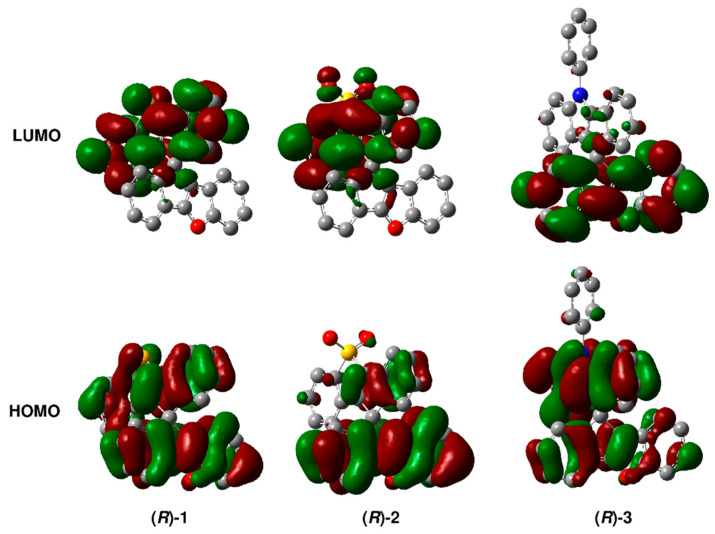
Frontier molecular orbitals of (*R*)-**1**–**3**.

In the PL spectra, *rac*-**1** exhibited emission maximum (λ_em_) at 369 nm in CH_2_Cl_2_ (Figure 3b). A slight red-shift was observed with an increase in solvent polarity (λ_em_: 361 nm (hexane), 366 nm (toluene), 366 nm (THF), 369 nm (CH_2_Cl_2_), and 381 nm (acetonitrile) Figure S19d). The *S*,*S*-dioxide derivative *rac*-**2** exhibited emission maximum at 434 nm in non-polar hexane, which is largely red-shifted compared to that of *rac*-**1**. With the increase in solvent polarity, a significant positive solvatochromic shift was observed (Figure 4a). In addition, the second emission band clearly appeared at the longer wavelength (519 nm) in CH_2_Cl_2_ and became predominant in the most polar acetonitrile (542 nm). According to our previous report [38], the shorter-wavelength emission band reflects the feature of the indeno[1,2-*b*][1]benzothiophene *S*,*S*-dioxide subunit. On the other hand, the longer-wavelength one could be ascribed to the photo-induced intramolecular charge transfer (ICT), in which the indeno[1,2-*b*][1]benzofuran and the indeno[1,2-*b*][1]benzothiophene *S*,*S*-dioxide subunits work as electron-donating and electron-accepting units, respectively. Indeed, the HOMO and LUMO estimated by DFT calculation are mainly localized in indeno[1,2-*b*][1]benzofuran and the indeno[1,2-*b*][1]benzothiophene *S*,*S*-dioxide subunits, respectively, by reflecting the donor–acceptor-type structure (Figure 5). The emission band of the pyrrole-containing compound *rac*-**3** is also red-shifted compared to that of *rac*-**1**. In addition, the PL spectrum of *rac*-**3** exhibited a stronger dependence on solvent polarity than that of *rac*-**1** (Figure 4b). The HOMO of *rac*-**3** is delocalized both on indeno[1,2-*b*][1]benzofuran and indeno[1,2-*b*]indole subunits, but the apparently larger distribution is demonstrated on the latter subunit. In contrast, the LUMO is mainly located on the indeno[1,2-*b*][1]benzofuran subunit. Therefore, indeno[1,2-*b*][1]benzofuran and indeno[1,2-*b*]indole subunits act as electron-accepting and electron-donating units, respectively, inducing an ICT character of *rac*-**3** in its emissive state and a clear positive solvatochromic shift in the PL spectrum.

Next, the effect of the furan unit on the emission properties of *rac*-**1**–**3** was evaluated through a comparison with those of the thiophene analogs **spiro-SS**, **SS(O)_2_**, and **-SN**. As described above, the PL spectrum of *rac*-**2** in CH_2_Cl_2_ clearly shows the longer-wavelength emission band, owing to a photo-induced ICT, and it is predominant in acetonitrile (Figure 4a). On the other hand, such an emission band is less clear and observed only as a shoulder in the case of **spiro-SS(O)_2_** [38]. This difference could be attributed to the more electron-rich furan unit, which works as a stronger electron-donating unit and allows more efficient photo-induced ICT. The degree of the solvatochromic shift of *rac*-**3** (λ_em_: 374 nm (hexane) and 421 (acetonitrile)) (Figure 4b) is slightly smaller than that of the thiophene analog **spiro-SN** (λ_em_: 375 nm (hexane) and 430 (acetonitrile)) [40]. The indeno[1,2-*b*]indole unit is the electron-donating unit both in *rac*-**3** and **spiro-SN**, and the indeno[1,2-*b*][1]benzofuran and the indeno[1,2-*b*][1]benzothiophene subunits work as electron-accepting units in *rac*-**3** and **spiro-SN**, respectively. Therefore, the smaller solvatochromic shift of *rac*-**3** could be due to the less efficient electron-accepting character of the more electron-rich indeno[1,2-*b*][1]benzofuran unit. The absolute quantum yields of *rac*-**1** and *rac*-**3** are 0.13 and 0.16, respectively (Table 1), which are much higher than those of **spiro-SS** (0.06) and **spiro-SN** (0.02) [38,40]. The *S*,*S*-dioxide derivative *rac*-**2** is merely emissive (<0.01), similar to **spiro-SS(O)_2_** [38].

**Figure 6:**
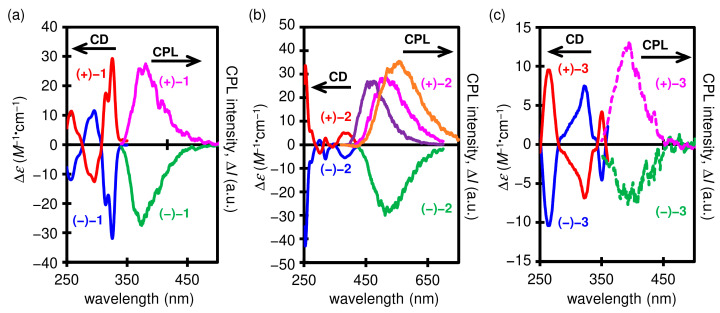
CD and CPL spectra of (**a**) **1**, (**b**) **2**, and (**c**) **3** in CH_2_Cl_2_ (red and blue lines for CD; pink and green lines for CPL; purple and orange lines in (**b**) for CPL in toluene and acetonitrile, respectively).

Chiroptical properties of **1**–**3** were investigated by CD and CPL spectroscopies (Figure 6 and Table 2). The CD spectrum of (+)-**1** exhibited two large positive Cotton effects at 340 nm and 328 nm and several negative and positive ones around 305 nm and 260 nm, respectively, which are quite similar to those of (+)-(*S*)-**spiro-SS** [38,39]. Therefore, the absolute configuration of (+)-**1** is considered to be *S*. The TD-DFT calculation results also support this assignment. CD spectra of (+)-**2** and (+)-**3** are slightly different from those of the thiophene analogs **spiro-SS(O)_2_** and **spiro-SN**, respectively, but the sign of the first Cotton effect of (+)-**2** and (+)-**3** is identical to that of (+)-(*S*)-isomers of **spiro-SS(O)_2_** and **spiro-SN** [38,40]. The dissymmetry factors in absorption, *g*_abs_ (Δ*ε*/*ε* = (*ε*_L_ − *ε*_R_)/[(*ε*_L_ + *ε*_R_)/2]), were estimated to be approximately 1.2 × 10^−3^ for (+)-**1**, 1.4 × 10^−3^ for (+)-**2**, and +1.4 × 10^−3^ for (+)-**3**. In the CPL spectra, (+)- and (−)-**1**–**3** gave mirror-image CPL spectra with each other. The dissymmetry factors in luminescence, *g*_lum_ [2Δ*I*/*I* = 2(*I*_L_ − *I*_R_)/(*I*_L_ + *I*_R_), *I*_L_ and *I*_R_: luminescence intensities of left and right circularly polarized light, respectively) [24], of (+)-**1**–**3** were estimated to be +0.90 × 10^−3^, +2.8 × 10^−3^, and +0.97 × 10^−3^, respectively, which are comparable to those of **spiro-SS**, **SS(O)_2_**, and **-SN [38,40]** and the previously reported chiral small organic molecules with significant CPL activity. The solvent effect on CPL was also investigated for (+)-**2,** since its PL spectrum exhibits significant positive solvatochromism. As expected, with the increase in solvent polarity, the CPL maximum shifted to the longer wavelength range (Figure 6b).

## 3. Materials and Methods

### 3.1. General Procedures

All manipulations that involved air- and/or moisture-sensitive compounds were carried out with the standard Schlenk technique under argon. Analytical thin-layer chromatography was performed on glass plates coated with 0.25-mm 230–400 mesh silica gel that contained a fluorescent indicator. Column chromatography was performed by using silica gel (spherical neutral, particle size 63–210 μm). The recycling preparative HPLC was performed with YMC–GPC T–2000 and T–4000 columns (chloroform as an eluent). Most of the reagents were purchased from commercial suppliers, such as Sigma-Aldrich Co. LLC (St. Louis, MO, USA), Tokyo Chemical Industry Co., Ltd. (Tokyo, Japan), and Kanto Chemical Co., Inc. (Tokyo, Japan), and used without further purification, unless otherwise specified. Commercially available anhydrous solvents were used for air- and/or moisture sensitive reactions. Compound **4** was prepared according to the literature [53].

NMR spectra were recorded in CDCl_3_ on a JEOL-ECX400 spectrometer (JEOL Ltd., Tokyo, Japan) (^1^H 400 MHz; ^13^C 101 MHz; ^19^F 376 MHz). Chemical shifts were reported in ppm relative to the internal standard signal (0 ppm for Me_4_Si in CDCl_3_ and acetone-*d*_6_) for ^1^H and the deuterated solvent signal (77.16 ppm for CDCl_3_ and 29.84 ppm for acetone-*d*_6_) for ^13^C. Data are presented as follows: chemical shift, multiplicity (s = singlet, brs = broad singlet, d = doublet, t = triplet, m = multiplet and/or multiple resonances), coupling constant in hertz (Hz), and signal area integration in natural numbers. Melting points were determined on SRS OptiMelt melting point apparatus (Stanford Research Systems, Sunnyvale, CA, USA). High resolution mass spectra were taken with a Bruker Daltonics micrOTOF–QII mass spectrometer (Bruker Corporation, Billerica, MA, USA) by the atmospheric pressure chemical ionization-time-of-flight (APCI–TOF) method. UV–vis absorption spectra were recorded on a JASCO V-650 spectrophotometer (JASCO Corporation, Tokyo, Japan). Photoluminescence spectra were recorded on a JASCO FP–6500 spectrofluorometer (JASCO Corporation, Tokyo, Japan). Absolute quantum yields were determined by an absolute quantum yield measurement system with a JASCO ILF–533 integrating sphere (JASCO Corporation, Tokyo, Japan). HPLC analyses and optical resolution were carried out using a DAICEL CHIRALPAK^®^ IA-3 column (4.6 mm × 250 mm) and a DAICEL CHIRALPAK^®^ IA column (20 mm × 250 mm) (Daicel Corporation, Tokyo, Japan), respectively. Circular dichroism (CD) spectra were recorded on a JASCO J–725 spectrometer (JASCO Corporation, Tokyo, Japan). CPL spectra were measured by using a JASCO CPL–300 spectrometer (JASCO Corporation, Tokyo, Japan). Optical rotations were measured on a JASCO P–2200 polarimeter (JASCO Corporation, Tokyo, Japan) using a 50-mm cell.

### 3.2. Synthesis

#### 3.2.1. Methyl 2-(1-Benzofuran-2-yl)benzoate (**5**)

A mixture of methyl 2-bromobenzoate (0.97 mL, 6.9 mmol), (1-benzofuran-2-yl)boronic acid (1.23 g, 7.6 mmol), Pd_2_(dba)_3_ (0.13 g, 0.14 mmol), SPhos (0.22 g, 0.53 mmol), and K_2_CO_3_ (2.86 g, 21 mmol) in anhydrous MeCN (30 mL) and deionized water (3 mL) was placed in a 100-mL Schlenk tube and degassed by three freeze–pump–thaw cycles. After stirring at 80 °C for 24 h under argon, the reaction mixture was cooled to room temperature. To the reaction mixture, 1 M aqueous HCl was slowly added, and the resulting mixture was extracted with CH_2_Cl_2_ (10 mL × 3). The combined organic layers were dried over anhydrous Na_2_SO_4_, filtered, and concentrated under reduced pressure. The resulting crude residue was purified by silica-gel column chromatography with hexane/CH_2_Cl_2_ (3/2, *R*_f_ = 0.48) as an eluent to give **5** as a colorless oil (1.55 g, 89% yield). The ^1^H and ^13^C NMR data were identical to those reported in the literature [54].


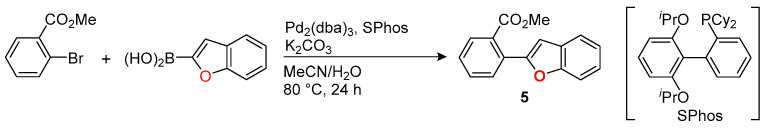


#### 3.2.2. *rac*-Spiro[indeno[1,2-*b*][1]benzofuran-10,10′-indeno[1,2-*b*][1]benzothiophene] (*rac*-**1**)

A mixture of **4** (0.74 g, 2.0 mmol) and *N*,*N*,*N*′,*N*′-tetramethylethylenediamine (1.2 mL, 8.0 mmol) in anhydrous THF (15 mL) was placed in a 50-mL Schlenk tube and cooled to −78 °C. To the mixture was added BuLi (2.3 M in hexane, 1.8 mL, 4.2 mmol) dropwise, and the resulting mixture was stirred at −78 °C for 1 h. Compound **5** (0.56 g, 2.2 mmol) in anhydrous THF (15 mL) was slowly added to the reaction mixture at −78 °C, and the resulting mixture was stirred at −78 °C for 1 h, warmed to ambient temperature, and then stirred for 18 h, before being quenched with water. The resulting mixture was extracted with EtOAc (5 mL × 3), and the combined organic layers were dried over anhydrous Na_2_SO_4_, filtered, and concentrated under reduced pressure. The resulting crude residue containing the tertiary alcohol **6** was used in the following step without purification.

The obtained crude residue was dissolved in CH_2_Cl_2_ (7 mL). To the solution, trifluoroacetic acid (1.5 mL) was added at ambient temperature, and the reaction mixture was stirred at ambient temperature for 6 h, before being quenched with aqueous saturated NaHCO_3_. The resulting mixture was extracted with CH_2_Cl_2_ (10 mL × 4), and the combined organic layers were dried over anhydrous Na_2_SO_4_, filtered, and concentrated under reduced pressure. The resulting crude residue was purified by silica-gel column chromatography with hexane (*R*_f_ = 0.38) as an eluent to give *rac*-**1** as a colorless solid (0.30 g, 37% yield); mp 245.5–246.6 °C; ^1^H NMR (400 MHz, CDCl_3_) δ 7.82 (d, *J* = 8.2 Hz, 1H), 7.74 (d, *J* = 7.3 Hz, 1H), 7.66 (d, *J* = 7.8 Hz, 1H), 7.58 (*d*, *J* = 8.2 Hz, 1H), 7.39–7.35 (m, 2H), 7.18–7.12 (m, 2H), 7.07 (td, *J* = 7.3, 0.9 Hz, H), 7.04 (td, *J* = 7.8, 0.9 Hz, H), 7.01–6.93 (m, 2H), 6.83 (d, *J* = 7.3 Hz, 1H), 6.77–6.73 (m, 3H); ^13^C NMR (101 MHz, CDCl_3_) δ 162.4, 160.2, 149.8, 148.7, 144.4, 144.0, 141.7, 139.2, 133.4, 128.3, 128.2, 127.2, 126.9, 125.5, 124.9, 124.4, 124.2, 124.0, 123.9, 123.8, 123.5, 123.3, 121.1, 120.1, 119.3, 118.2, 112.4, 57.4 (one missing signal for an aromatic carbon is presumed to overlap one of the signals that was observed.); HRMS–APCI^+^ (*m*/*z*) calcd for C_29_H_17_OS^+^ ([M + H]^+^), 413.0995, found 413.0994. 


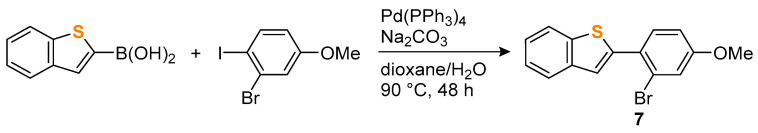


#### 3.2.3. 2-(2-Bromo-4-methoxyphenyl)-1-benzothiophene (**7**)

A mixture of (1-benzothiophen-2-yl)boronic acid (99 mg, 0.55 mmol), 2-bromo-1-iodo-4-methoxybenzene (0.15 g, 0.49 mmol), Na_2_CO_3_ (0.11 g, 1.1 mmol), and Pd(PPh_3_)_4_ (29 mg, 25 μmol) in anhydrous 1,4-dioxane (2.1 mL) and deionized water (0.30 mL) was placed in a 30-mL Schlenk tube and degassed by three freeze–pump–thaw cycles. After stirring at 90 °C for 48 h under argon, the reaction mixture was cooled to room temperature. To the reaction mixture, 1 M aqueous HCl was slowly added, and the resulting mixture was extracted with CH_2_Cl_2_ (5 mL × 3). The combined organic layers were dried over anhydrous Na_2_SO_4_, filtered, and concentrated under reduced pressure. The resulting crude residue was purified by silica-gel column chromatography with hexane/EtOAc (9/1, *R*_f_ = 0.52) as an eluent and preparative HPLC to give **7** as a colorless solid (0.11 g, 67% yield): mp 82.3–84.1 °C; ^1^H NMR (400 MHz, CDCl_3_) *δ* 7.84 (dd, *J* = 8.2, 0.9 Hz, 1H), 7.80 (dd, *J* = 7.3, 1.4 Hz, 1H), 7.46 (d, *J* = 8.2 Hz, 1H), 7.42 (s, 1H), 7.37 (td, *J* = 6.9, 1.4 Hz, 1H), 7.32 (td, *J* = 7.3, 1.4 Hz, 1H), 7.25 (d, *J* = 2.7 Hz, 1H), 6.92 (dd, *J* = 8.7, 2.7 Hz, 1H), 3.85 (s, 3H); ^13^C NMR (101 MHz, CDCl_3_) δ 160.0, 142.1, 140.2, 140.0, 132.9, 127.8, 124.5, 124.4, 124.2, 123.8, 123.6, 122.2, 118.9, 113.8, 55.8; HRMS–APCI^+^ (*m*/*z*) calcd for C_15_H_12_BrOS^+^ ([M + H]^+^), 318.9787, found 318.9791.


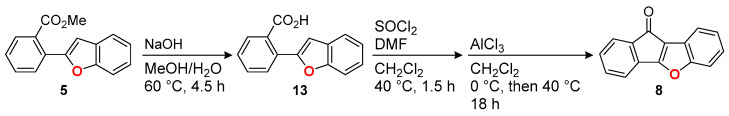


#### 3.2.4. 2-(1-Benzofuran-2-yl)benzoic Acid (**13**)

To a solution of **5** (1.75 g, 6.9 mmol) in methanol (20 mL), a mixture of NaOH (1.68 g, 42 mmol) and water (20 mL) was added at room temperature. After stirring at 60 °C for 4.5 h, the reaction mixture was cooled to room temperature and acidified with 1 M aqueous HCl. The resulting mixture was extracted with Et_2_O (10 mL × 4). The combined organic layers were dried over anhydrous Na_2_SO_4_, filtered, and concentrated under reduced pressure to give **5** as a colorless solid (1.60 g, 97% yield); mp 132.8–135.3 °C; ^1^H NMR (400 MHz, acetone-*d*_6_) δ 11.30 (br, 1H), 7.84 (d, *J* = 7.3 Hz, 1H), 7.83 (d, *J* = 7.3 Hz, 1H), 7.67–7.62 (m, 2H), 7.55 (td, *J* = 7.8, 1.4 Hz, 1H), 7.51 (ddd, *J* = 8.2, 1.8, 0.9 Hz, 1H), 7.3 (ddd, *J* = 8.7, 7.3, 1.4 Hz, 1H), 7.25 (td, *J* = 7.6, 1.4 Hz, 1H), 7.12 (d, *J* = 0.9 Hz, 1H); ^13^C NMR (101 MHz, acetone-*d*_6_) δ 169.6, 155.93, 155.88, 132.8, 131.9, 130.4, 130.2, 130.1, 130.0, 129.7, 125.3, 123.8, 122.1, 111.8, 105.3; HRMS–APCI^+^ (*m*/*z*) calcd for C_15_H_11_O_3_^+^ ([M + H]^+^), 239.0703, found 239.0702.

#### 3.2.5. 10*H*-Indeno[1,2-*b*][1]benzofuran-10-one (**8**)

A mixture of **13** (3.83 g, 16 mmol) and thionyl chloride (1.4 mL, 19 mmol) in anhydrous CH_2_Cl_2_ (160 mL) was placed in a 300-mL three-neck flask with a condenser at ambient temperature. To the mixture, anhydrous DMF (100 µL) was added, and the resulting mixture was stirred for 1.5 h at 40 °C. The volatiles were removed under reduced pressure to afford the acid chloride intermediate.

A mixture of AlCl_3_ (3.26 g, 24 mmol) and anhydrous CH_2_Cl_2_ (180 mL) was charged in a 500-mL three-neck flask with a condenser and a dropping funnel and cooled at 0 °C. To the mixture, a solution of the acid chloride intermediate in anhydrous CH_2_Cl_2_ (160 mL) was slowly added via the dropping funnel over 2 h at 0 °C under argon atmosphere. The reaction mixture was stirred for 18 h at 40 °C, cooled to ambient temperature, and then poured into ice-cooled 1 M aqueous HCl. The resulting mixture was extracted with CH_2_Cl_2_ (100 mL × 3), and the combined organic layers were dried over anhydrous Na_2_SO_4_, filtered, and concentrated under reduced pressure. The resulting crude residue was purified by silica-gel column chromatography with hexane/EtOAc (9/1, *R*_f_ = 0.49) as an eluent to give **8** as an orange solid (1.44 g, 41% yield). The ^1^H and ^13^C NMR data were identical to those reported in the literature [55].

#### 3.2.6. *rac*-2′-Methoxyspiro[indeno[1,2-*b*][1]benzofuran-10,10′-indeno[1,2-*b*][1]benzothiophene] (*rac*-**10**)

A mixture of **7** (0.48 g, 1.5 mmol) and *N*,*N*,*N*′,*N*′-tetramethylethylenediamine (0.45 mL, 3.0 mmol) in anhydrous THF (12 mL) was placed in a 100-mL Schlenk flask and cooled to −78 °C. To the mixture, BuLi (2.6 M in hexane, 0.59 mL, 1.5 mmol) was added dropwise, and the resulting mixture was stirred at −78 °C for 1 h. Compound **8** (0.27 g, 1.3 mmol) in anhydrous THF (12 mL) was slowly added to the reaction mixture at −78 °C, and the resulting mixture was stirred at −78 °C for 1 h, warmed to ambient temperature, and then stirred for 18 h, before being quenched with water. The resulting mixture was extracted with CH_2_Cl_2_ (10 mL × 3), and the combined organic layers were dried over anhydrous Na_2_SO_4_, filtered, and concentrated under reduced pressure. The resulting crude residue was roughly purified by silica-gel column chromatography with hexane/EtOAc (9/1) as an eluent. The fractions (*R*_f_ = 0.16) that contained the tertiary alcohol **9** and some impurities were concentrated under reduced pressure.

The resulting residue was dissolved in CH_2_Cl_2_ (10 mL) and cooled to 0 °C. To the solution, trifluoroacetic acid (0.4 mL) was added at 0 °C, and the reaction mixture was stirred at 0 °C for 1 h, before being quenched with aqueous saturated NaHCO_3_. The resulting mixture was extracted with CH_2_Cl_2_ (10 mL × 3), and the combined organic layers were dried over anhydrous Na_2_SO_4_, filtered, and concentrated under reduced pressure. The resulting crude residue was purified by silica-gel column chromatography with hexane/EtOAc (9/1, *R*_f_ = 0.49) as an eluent to give *rac*-**10** as a colorless solid (0.37 g, 66% yield from **8**); mp 216.4–221.6 °C; ^1^H NMR (400 MHz, CDCl_3_) δ 7.78 (ddd, *J* = 8.2, 1.8, 0.9 Hz, 1H), 7.73 (ddd, *J* = 7.3, 1.8, 0.9 Hz, 1H), 7.57 (d, *J* = 8.2, 1H), 7.55 (d, *J* = 8.2, 1H), 7.36 (td, *J* = 7.6, 0.9 Hz, 1H), 7.15 (ddd, *J* = 8.2, 6.9, 1.4, 1H), 7.10 (ddd, *J* = 8.2, 6.9, 1.4, 1H), 7.04 (td, *J* = 7.6, 1.4 Hz, 1H), 6.98–6.94 (m, 2H), 6.89 (dd, *J* = 8.2, 2.3 Hz, 1H), 6.79–6.77 (m, 2H), 6.69 (ddd, *J* = 7.8, 1.4, 0.9 Hz, 1H), 6.39 (d, *J* = 2.3 Hz, 1H), 3.63 (s, 3H); ^13^C NMR (101 MHz, CDCl_3_) δ 162.3, 160.2, 159.4, 150.9, 150.1, 144.4, 143.5, 140.1, 133.7, 133.4, 132.2, 128.2, 127.2, 125.9, 124.8, 124.4, 124.1, 123.9, 123.7, 123.6, 123.5, 120.7, 120.6, 119.4, 118.1, 113.4, 112.4, 109.9, 57.4, 55.6; HRMS–APCI^+^ (*m*/*z*) calcd for C_30_H_19_O_2_S^+^ ([M + H]^+^), 443.1100, found 443.1103.

#### 3.2.7. *rac*-Spiro[indeno[1,2-*b*][1]benzofuran-10,10′-indeno[1,2-*b*][1]benzothiophene]-2′-ol (*rac*-**11**)

To a mixture of *rac*-**10** (1.04 g, 2.4 mmol,) and anhydrous CH_2_Cl_2_ (24 mL), BBr_3_ (1 M in CH_2_Cl_2_, 4.7 mL, 4.7 mmol) was added dropwise at 0 °C. After stirring at 0 °C for 2.5 h, the reaction was quenched with water. The resulting mixture was extracted with CH_2_Cl_2_ (15 mL × 3) and the combined organic layers were dried over anhydrous Na_2_SO_4_, filtered, and concentrated under reduced pressure. The resulting crude residue was purified by silica-gel column chromatography with hexane/EtOAc (3/1, *R*_f_ = 0.46) as an eluent to give *rac*-**11** as a colorless solid (0.59 g, 58% yield). *rac*-**11** can be separated into enantiopure (−)-**11** and (+)-**11** by HPLC equipped with an analytical DAICEL CHIRALPAK^®^ IA-3 column (Φ4.6 mm × 250 mm) (*t*_R_ = 12.7 min for (−)-**11** and 14.5 min for (+)-**11** (flow rate: 1.0 mL/min; eluent: hexane/CHCl_3_ = 50/50)): [α]_D_^25^ for (+)-**11** +175 (*c* 0.112, CH_2_Cl_2_); mp 261.2–265.7 °C; ^1^H NMR (500 MHz, CDCl_3_) *δ* 7.80 (d, *J* = 8.0 Hz, 1H), 7.74 (d, *J* = 7.4 Hz, 1H), 7.58 (d, *J* = 8.6 Hz, 1H), 7.52 (d, *J* = 8.0 Hz, 1H), 7.38 (t, *J* = 7.2 Hz, 1H), 7.18 (td, *J* = 8.0, 1.2 Hz, 1H), 7.12 (td, *J* = 8.0, 1.2 Hz, 1H), 7.06 (td, *J* = 7.5, 1.2 Hz, 1H), 7.00–6.96 (m, 2H), 6.84 (dd, *J* = 8.0, 2.3 Hz, 1H), 6.80–6.79 (m, 2H), 6.70 (d, *J* = 8.0 Hz, 1H), 6.32 (d, *J* = 2.6 Hz, 1H), 4.54 (brs, 1H); ^13^C NMR (101 MHz, CDCl_3_) δ 162.3, 160.2, 155.1, 151.1, 150.0, 144.4, 143.5, 140.0, 133.6, 133.3, 132.3, 128.2, 127.2, 125.7, 124.8, 124.4, 124.1, 124.0, 123.75, 123.69, 123.5, 120.9, 120.7, 119.3, 118.2, 115.0, 112.4, 111.2, 57.3; HRMS–APCI^+^ (*m*/*z*) calcd for C_29_H_17_O_2_S^+^ ([M + H]^+^), 429.0944, found 429.0931.

#### 3.2.8. *rac*-Spiro[indeno[1,2-*b*][1]benzofuran-10,10′-indeno[1,2-*b*][1]benzothiophene]-2′-yl Trifluoromethanesulfonate (*rac*-**12**)

A mixture of *rac*-**11** (82 mg, 0.19 mmol) and anhydrous triethylamine (0.14 mL, 1.0 mmol) in anhydrous CH_2_Cl_2_ (2 mL) was placed in a 20-mL Schlenk tube and cooled to –78 °C. To the mixture, trifluoromethanesulfonic anhydride (45 µL, 0.27 mmol) was added dropwise, and the resulting mixture was stirred at –78 °C for 30 min. The reaction was quenched with water, and then the resulting mixture was extracted with CH_2_Cl_2_ (5 mL × 3). The combined organic layers were dried over anhydrous Na_2_SO_4_, filtered, and concentrated under reduced pressure. The resulting crude residue was purified by silica-gel column chromatography with hexane/EtOAc (9/1, *R*_f_ = 0.43) as an eluent to give *rac*-**12** as a colorless solid (92 mg, 85% yield); ^1^H NMR (400 MHz, CDCl_3_) *δ* 7.84 (d, *J* = 7.8 Hz, 1H), 7.77 (d, *J* = 7.3 Hz, 1H), 7.70 (d, *J* = 8.2 Hz, 1H), 7.60 (d, *J* = 8.2 Hz, 1H), 7.42 (dd, *J* = 7.6, 6.6 Hz, 1H), 7.32 (dd, *J* = 8.2, 2.3 Hz, 1H), 7.22–7.18 (m, 2H), 7.09 (td, *J* = 7.6, 0.9 Hz, 1H), 7.02 (td, *J* = 7.3, 0.9 Hz, 1H), 6.99 (t, *J* = 7.8 Hz, 1H), 6.77–6.72 (m, 4H); ^13^C NMR (101 MHz, CDCl_3_) δ 162.6, 160.2, 151.5, 148.3, 144.4, 143.7, 142.4, 139.5, 133.3, 133.1, 128.7, 127.5, 125.2, 124.9, 124.3, 124.2, 124.0, 123.92, 123.89, 123.7, 121.5, 121.4, 120.8, 119.0, 118.70 (q, *J*_CF_ = 321 Hz), 118.5, 116.9, 112.6, 57.5 (one missing signal for an aromatic carbon is presumed to overlap one of the signals that was observed.); ^19^F NMR (376 MHz, CDCl_3_) δ − 72.8; HRMS–APCI^+^ (*m*/*z*) calcd for C_30_H_16_F_3_O_4_S_2_^+^ ([M + H]^+^), 561.0437, found 561.0443.

The crude product of (+)-**12** was also obtained by using (+)-**11** (62 mg, 0.14 mmol), anhydrous triethylamine (0.10 mL, 0.72 mmol), and trifluoromethanesulfonic anhydride (32 µL, 0.20 mmol), according to the procedure for *rac*-**12**. Purification by silica-gel column chromatography with hexane/EtOAc (9/1, *R*_f_ = 0.43) as an eluent gave (+)-**12** as a colorless solid (70 mg, 87% yield): [α]_D_^25^ +128 (*c* 0.158, CH_2_Cl_2_). The ^1^H and ^13^C NMR spectra were identical to those of *rac*-**12**.

#### 3.2.9. *rac*-Spiro[indeno[1,2-*b*][1]benzofuran-10,10′-indeno[1,2-*b*][1]benzothiophene] (*rac*-**1**)

A mixture of *rac*-**12** (56 mg, 0.10 mmol), Pd(OAc)_2_ (1.2 mg, 5.3 µmol), PPh_3_ (2.4 mg, 9.2 µmol), anhydrous triethylamine (85 µL, 0.61 mmol), formic acid (15 µL, 0.39 mmol), and anhydrous DMF (1.0 mL) was placed in a 20-mL Schlenk tube and degassed by three freeze–pump–thaw cycles. After stirring at 80 °C for 2 h under argon, the reaction mixture was cooled to room temperature. The reaction was quenched by adding 1 M aqueous HCl slowly, and the resulting mixture was extracted with CH_2_Cl_2_ (5.0 mL × 3). The combined organic layers were dried over anhydrous Na_2_SO_4_, filtered, and concentrated under reduced pressure. The resulting crude residue was purified by silica-gel column chromatography with hexane/EtOAc (9/1, *R*_f_ = 0.70) as an eluent to give *rac*-**1** as a colorless solid (40 mg, 96% yield). The ^1^H and ^13^C NMR spectra were identical to those of *rac*-**1** described above.

The crude product of (+)-**1** was also obtained by using (+)-**12** (79 mg, 0.12 mmol), Pd(Oac)_2_ (1.4 mg, 6.2 μmol), PPh_3_ (3.1 mg, 12 μmol), anhydrous trimethylamine (0.12 mL, 0.83 mmol), formic acid (22 μL, 0.53 mmol), and DMF (1.4 mL), according to the procedure for *rac*-**1**. Purification by silica-gel column chromatography with hexane/EtOAc (9/1, *R*_f_ = 0.70) as an eluent gave (+)-**1** as a colorless solid (52 mg, 89% yield): [α]_D_^25^ +154 (*c* 0.298, CH_2_Cl_2_). The ^1^H and ^13^C NMR spectra were identical to those of *rac*-**1**.

#### 3.2.10. *rac*-Spiro[indeno[1,2-*b*][1]benzofuran-10,10′-indeno[1,2-*b*][1]benzothiophene] 5′,5′-Dioxide (*rac*-**2**)

To a mixture of *rac*-**1** (0.12 g, 0.30 mmol) and CH_2_Cl_2_ (2.0 mL), *m*-chloroperoxybenzoic acid (contains ca. 30% water, 0.19 g, 1.1 mmol) was added at 0 °C. The resulting mixture was stirred at 0 °C for 24 h. The reaction was quenched with a mixture of an aqueous saturated Na_2_S_2_O_3_ and NaHCO_3_, and then the resulting mixture was extracted with CH_2_Cl_2_ (5.0 mL × 3). The combined organic layers were dried over anhydrous Na_2_SO_4_, filtered, and concentrated under reduced pressure. The resulting crude residue was purified by silica-gel column chromatography with CHCl_3_ (*R*_f_ = 0.81) as an eluent to give *rac*-**2** as a colorless solid (0.13 mg, 96% yield); ^1^H NMR (400 MHz, CDCl_3_) *δ* 7.83 (d, *J* = 7.8 Hz, 1H), 7.77 (d, *J* = 7.3 Hz, 1H), 7.66 (d, *J* = 7.3 Hz, 1H), 7.59 (d, *J* = 8.2 Hz, 1H), 7.46–7.41 (m, 2H), 7.28 (t, *J* = 7.6 Hz, 1H), 7.24–7.20 (m, 1H), 7.18–7.09 (m, 3H), 7.06 (t, *J* = 7.6 Hz, 1H), 6.94 (m, 2H), 6.84 (d, *J* = 7.8 Hz, 1H), 6.35 (d, *J* = 7.3 Hz, 1H); ^13^C NMR (101 MHz, CDCl_3_) δ 162.8, 160.2, 151.4, 147.7, 146.3, 144.5, 142.9, 134.0, 133.5, 133.3, 130.0, 129.2, 128.9, 128.5, 128.0, 127.8, 124.6, 124.3, 124.0, 123.8, 123.7, 122.9, 121.8, 121.6, 119.2, 118.8, 112.6, 57.7 (one missing signal for an aromatic carbon is presumed to overlap one of the signals that was observed.); HRMS–APCI^+^ (*m/z*) calcd for C_29_H_17_O_3_S^+^ ([M + H]^+^), 445.0893, found 445.0891.

The crude product of (+)-**2** was obtained by using (+)-**1** (27 mg, 65 μmol), *m*-chloroperoxybenzoic acid (41 mg, 0.17 mmol), and CH_2_Cl_2_ (0.7 mL), according to the procedure for *rac*-**2**. Purification by silica-gel column chromatography with CHCl_3_ (*R*_f_ = 0.81) as an eluent gave (+)-**2** as a colorless solid (28 mg, 96% yield): [α]_D_^25^ +151 (*c* 0.15, CH_2_Cl_2_). The ^1^H and ^13^C NMR spectra were identical to those of *rac*-**2**.

#### 3.2.11. *rac*-5′-Phenyl-5′*H*-spiro[indeno[1,2-*b*][1]benzofuran-10,10′-indeno[1,2-*b*]indole] (*rac*-**3**)

A solution of KHMDS (0.5 M in toluene, 0.19 mL, 93 μmol) was charged in a 20-mL Schlenk tube, and toluene was removed under reduced pressure. The resulting residue was dissolved with anhydrous xylene (0.3 mL) under argon. The resulting xylene solution of KHMDS was added to a mixture of *rac*-**2** (14 mg, 31 μmol) and aniline (5.7 µL, 62 μmol) in another 20-mL Schlenk tube under argon at ambient temperature. The resulting solution was stirred at 140 °C for 48 h. The reaction mixture was cooled to an ambient temperature, and 1 M aqueous HCl was added slowly to the reaction mixture. The resulting mixture was extracted with CH_2_Cl_2_ (5 mL × 4), and the combined organic layers were dried over Na_2_SO_4_, filtered, and concentrated under reduced pressure. The crude residue was purified by silica-gel column chromatography with hexane/EtOAc (9/1, *R*_f_ = 0.48) as an eluent to give *rac*-**3** as a colorless solid (9.4 mg, 64% yield); mp 255.1–259.2 °C; ^1^H NMR (400 MHz, CDCl_3_) *δ* 7.75–7.66 (m, 5H), 7.60–7.54 (m, 2H), 7.40–7.34 (m, 2H), 7.18–7.13 (m, 3H), 7.08–7.03 (m, 2H), 7.00–6.94 (m, 2H), 6.89–6.78 (m, 5H); ^13^C NMR (101 MHz, CDCl_3_) δ 162.3, 160.2, 151.3, 150.6, 144.6, 142.5, 138.1, 135.4, 133.5, 129.8, 128.0, 127.8, 127.5, 127.1, 127.0, 126.4, 126.2, 124.7, 123.8, 123.69, 123.67, 123.34, 123.29, 122.8, 122.3, 120.9, 119.4, 119.0, 118.9, 117.9, 112.3, 111.1, 54.3; HRMS–APCI^+^ (*m/z*) calcd for C_35_H_22_NO^+^ ([M + H]^+^), 472.1696, found 472.1687.

The crude product of (+)-**3** was obtained by using (+)-**2** (16 mg, 36 μmol), aniline (7 μL, 77 μmol), KHMDS (0.5 M in toluene, 0.22 mL), and xylene (0.36 mL), according to the procedure for *rac*-**3**. Purification by silica-gel column chromatography with hexane/EtOAc (9/1, *R*_f_ = 0.48) as an eluent gave (+)-**3** as a colorless solid (4.7 mg, 27% yield): [α]_D_^25^ +175 (*c* 0.094, CH_2_Cl_2_). The ^1^H and ^13^C NMR spectra were identical to those of *rac*-**3**.

### 3.3. Computational Studies

The DFT and TD-DFT calculations were performed by using the Gaussian 09 program [56] at the B3LYP/6-31G(d) level of theory in the gas phase. The starting molecular models for DFT geometry optimizations were built and optimized with MMFF molecular mechanics by using the Spartan ’08 package (Wavefunction, Inc., Irvine, CA, USA). Six singlet states were calculated in the TD-DFT calculations. The visualization of the molecular orbitals was performed using GaussView 5.

### 3.4. X-ray Crystallography

For X-ray crystallographic analysis, a suitable single crystal was selected under ambient conditions, mounted using a nylon loop filled with paraffin oil, and transferred to the goniometer of a RIGAKU R-AXIS RAPID diffractometer (Rigaku Corporation, Tokyo, Japan), with graphite-monochromated Cu–Kα irradiation (λ = 1.54187 Å). The structure was solved by a direct method (SIR 2008 [57]) and refined by full-matrix least-squares techniques against *F*^2^ (SHELXL-2014 [58,59]). The intensities were corrected for Lorentz and polarization effects. All non-hydrogen atoms were refined anisotropically. Hydrogen atoms were placed using AFIX instructions.

Crystal data for *rac*-**1**: formula: C_29_H_16_OS (*M* = 412.48 g/mol): monoclinic, space group *P*2_1_/*n* (No. 14), *a* = 14.2753(3) Å, *b* = 9.8401(2) Å, *c* = 15.7643(3) Å, *β* = 114.4630(10)°, *V* = 2015.63(7) Å^3^, *Z* = 4, *T* = 193(2) K, *μ*(CuKα) = 1.566 mm^−1^, *D*_calc_ = 1.359 g/cm^3^, 34,993 reflections measured (3.519° ≤ *θ* ≤ 68.226°), 3684 unique (*R*_int_ = 0.0448; *R*_sigma_ = 0.0213), which were used in all calculations. The final *R*_1_ was 0.0688 (*I* > 2σ(*I*)) and w*R*_2_ was 0.2028 (all data). CCDC 2,191,229 contains the supplementary crystallographic data for this paper. The data can be obtained free of charge from the Cambridge Crystallographic Data Centre via www.ccdc.cam.ac.uk/structures, accessed on 8 August 2022.

## 4. Conclusions

In summary, we have synthesized furan-containing chiral spiro-fused PACs **1–3**. Aromatic metamorphosis strategy was applied to **1**, affording the pyrrole-containing **3,** as well as the *S*,*S*-dioxide derivative **2**. The hydroxylated derivatives of **1** were found to be separated into enantiopure isomers by chiral HPLC, which allowed us to prepare enantiomers of **1–3**. Introduction of a furan unit has proven to have a great impact on their photophysical properties. The observed impact could be attributed to the increased electron-richness of furan compared to thiophene. The synthesized furan-containing chiral spiro-fused PACs demonstrated CPL with a *g*_lum_ value of <2.3 × 10^−3^, which is comparable to those of the reported chiral small organic molecules.

## Figures and Tables

**Table 1:** Photophysical properties of spiro-fused PACs **1**–**3**.

	λ_abs_ (nm) *^a^*	λ_em_ (nm) *^b^*	Φ *^c^*
*rac*-**1**	337	369 (320)	13
*rac*-**2**	332, 355 (sh)	480, 519 (330)	<1
*rac*-**3**	335, 353 (sh)	402 (330)	16
*rac*-**SS***^d^*	340	368 (330)	6
*rac*-**SS(O)_2_***^d^*	336, 356 (sh)	459 (380)	1
*rac*-**SN***^e^*	334, 350 (sh)	414 (330)	2

*^a^* The longest absorption maximum in CH_2_Cl_2_. sh: shoulder. *^b^* Emission maximum in CH_2_Cl_2_. Excitation wavelength in parenthesis. *^c^* Absolute quantum yield in CH_2_Cl_2_. *^d^* Reference [38]. *^e^* Reference [40].

**Table 2:** Chiroptical date of spiro-fused PACs **1**–**3** in CH_2_Cl_2_.

	*g_abs_* (×10^−3^) *^a^*	*g_lum_* (×10^−3^) *^b^*	|[α]_D_^25^| *^c^*
(+)-**1**	+1.2 (341 nm)	+0.90 (381 nm)	154 (*c* 0.298)
(+)-**2**	+1.4 (378 nm)	+2.8 (517 nm)	151 (*c* 0.150)
(+)-**3**	+0.31 (351 nm)	+0.97 (394 nm)	175 (*c* 0.094)

*^a^* Calculated at the longest CD maximum. Wavelength for the calculated *g_abs_* in parentheses. *^b^* Calculated at the CPL maximum. Wavelength for the calculated *g_lum_* in parentheses. *^c^* In CH_2_Cl_2_. Concentration in parentheses.

## Data Availability

The data presented in this study are available in the Supplementary Materials.

## Supplementary Materials

The following supporting information can be downloaded at: https://www.mdpi.com/article/10.3390/molecules27165103/s1, Figures S1–S17: ^1^H, ^13^C, and ^19^F NMR spectra for **1**–**3**, **7**, and **10**–**13**; Table S1: Crystallographic data for *rac*-**1**; Figure S18: HPLC chart for optical resolution of **11**; Figure S19: UV–vis (**1**–**3**) and PL (**1**) spectra in various solvents; Figures S20–S22: Molecular orbitals for (*R*)-**1**–**3**; Tables S2–S4: Coordinates and absolute energy of the optimized structures for (*R*)-**1**–**3**; Table S5: The selected absorption of (*R*)-**1**–**3** calculated by TD-DFT method.

## Sample Availability

Samples of the compounds are available from the authors.
