# Supplementary material for: Furan-Containing Chiral Spiro-Fused Polycyclic Aromatic Compounds: Synthesis and Photophysical Properties
Source: Molecules. 2022 Aug 11;27(16):5103. doi: 10.3390/molecules27165103 (PMC9415352; doi:10.3390/molecules27165103)
Supplement: Supplementary file 1 [file molecules-27-05103-s001.zip › molecules-1854551-supplementary.pdf]

Supporting Information

Furan-Containing Chiral Spiro-Fused Polycyclic Aromatic Compounds: Synthesis and Photophysical Properties

Koji Nakano,*,† Ko Takase,† Keiichi Noguchi‡

†Department of Organic and Polymer Materials Chemistry, and ‡Instrumentation Analysis Center, Tokyo University of Agriculture and Technology, 2-24-16 Naka-cho, Koganei, Tokyo 184-8588, Japan

e-mail: k_nakano@cc.tuat.ac.jp

Table of Contents

^{1}H ^{13}C, and ^{19}F Spectra of Compounds S2–S10

X-ray Analysis S11

Chiral HPLC Chart and UV–vis/PL Spectra S12

Theoretical Calculation Results S13–S19

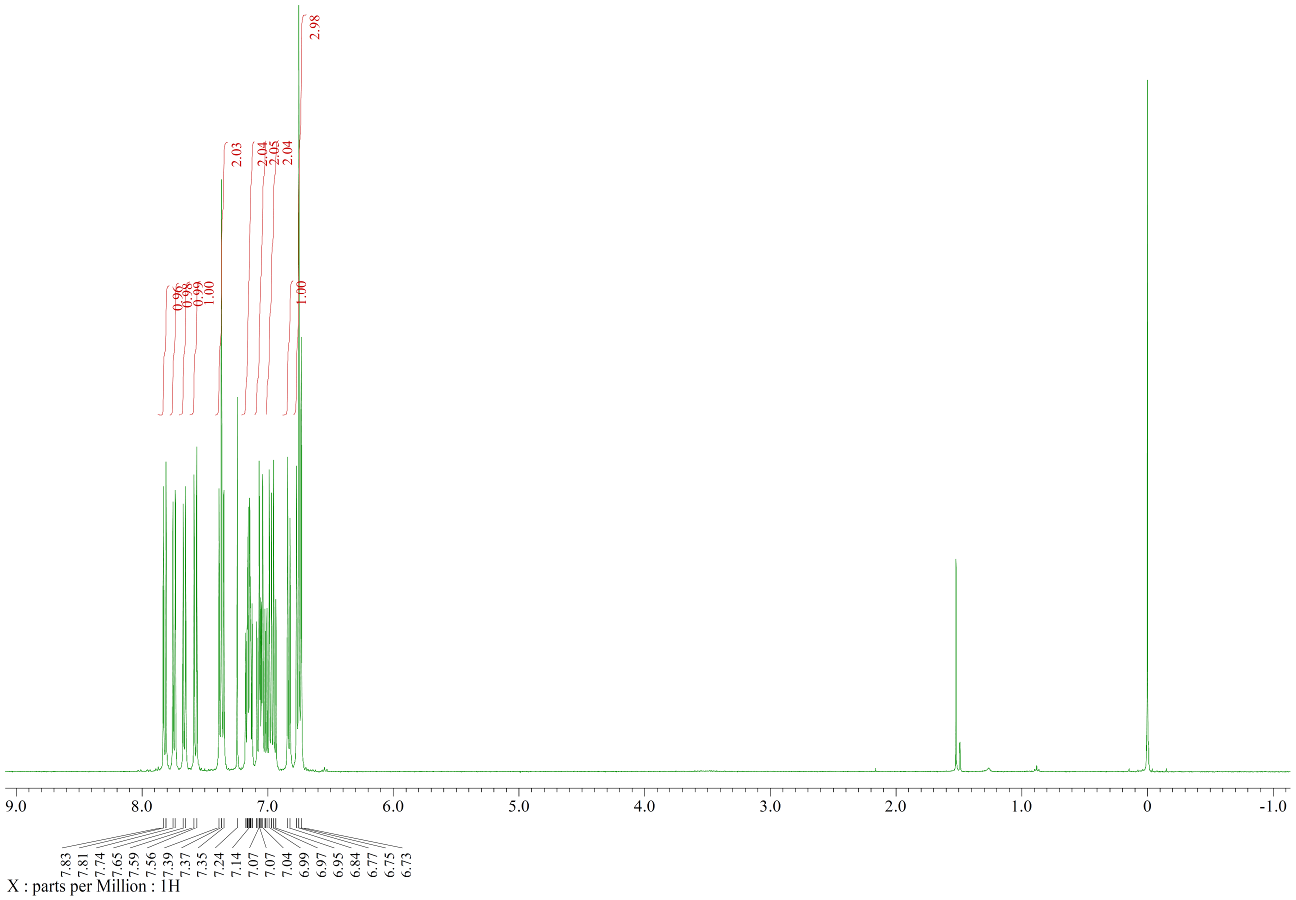

Figure S1. ^{1}H NMR spectrum of *rac*-**1** (400 MHz, $CDCl_3$).

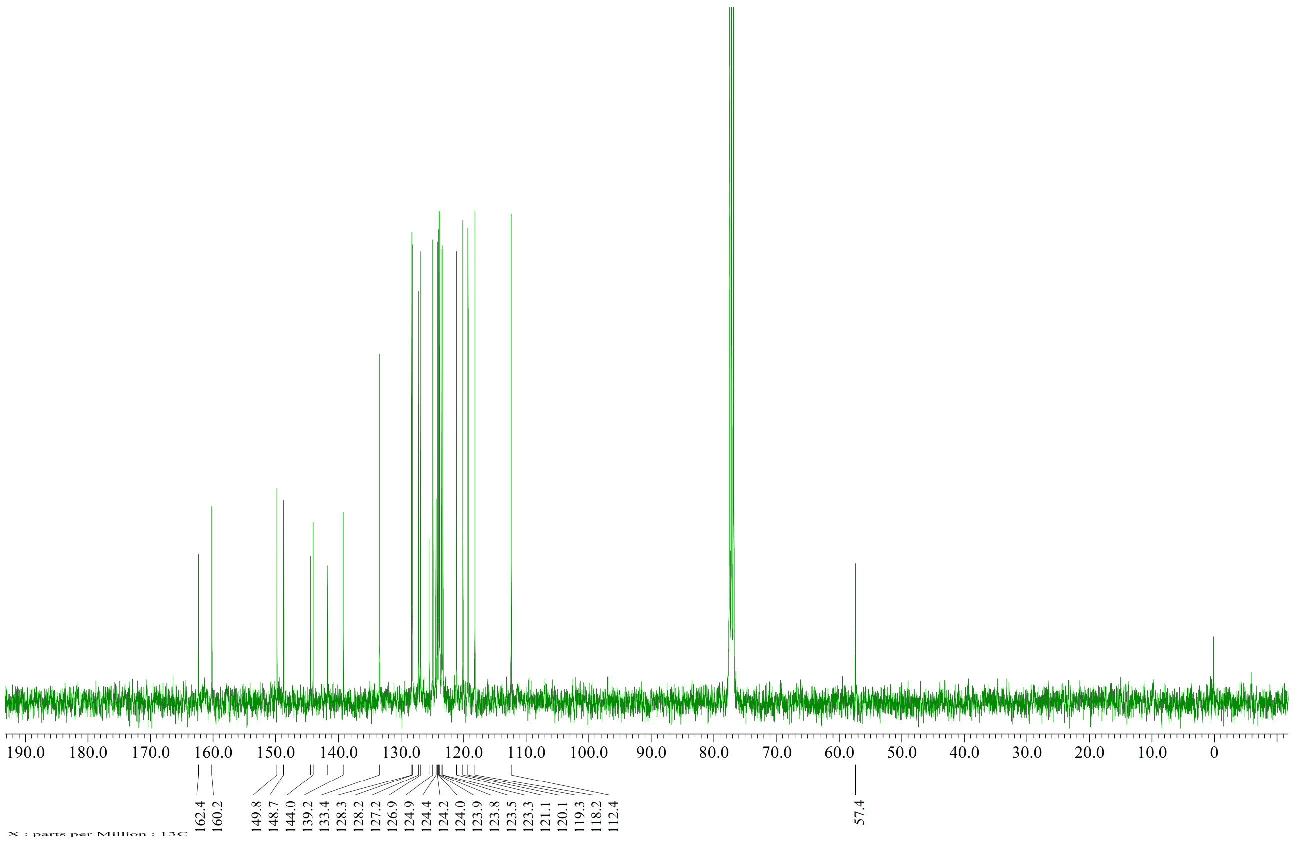

Figure S2. ^{13}C NMR spectrum of *rac*-**1** (101 MHz, $CDCl_3$).

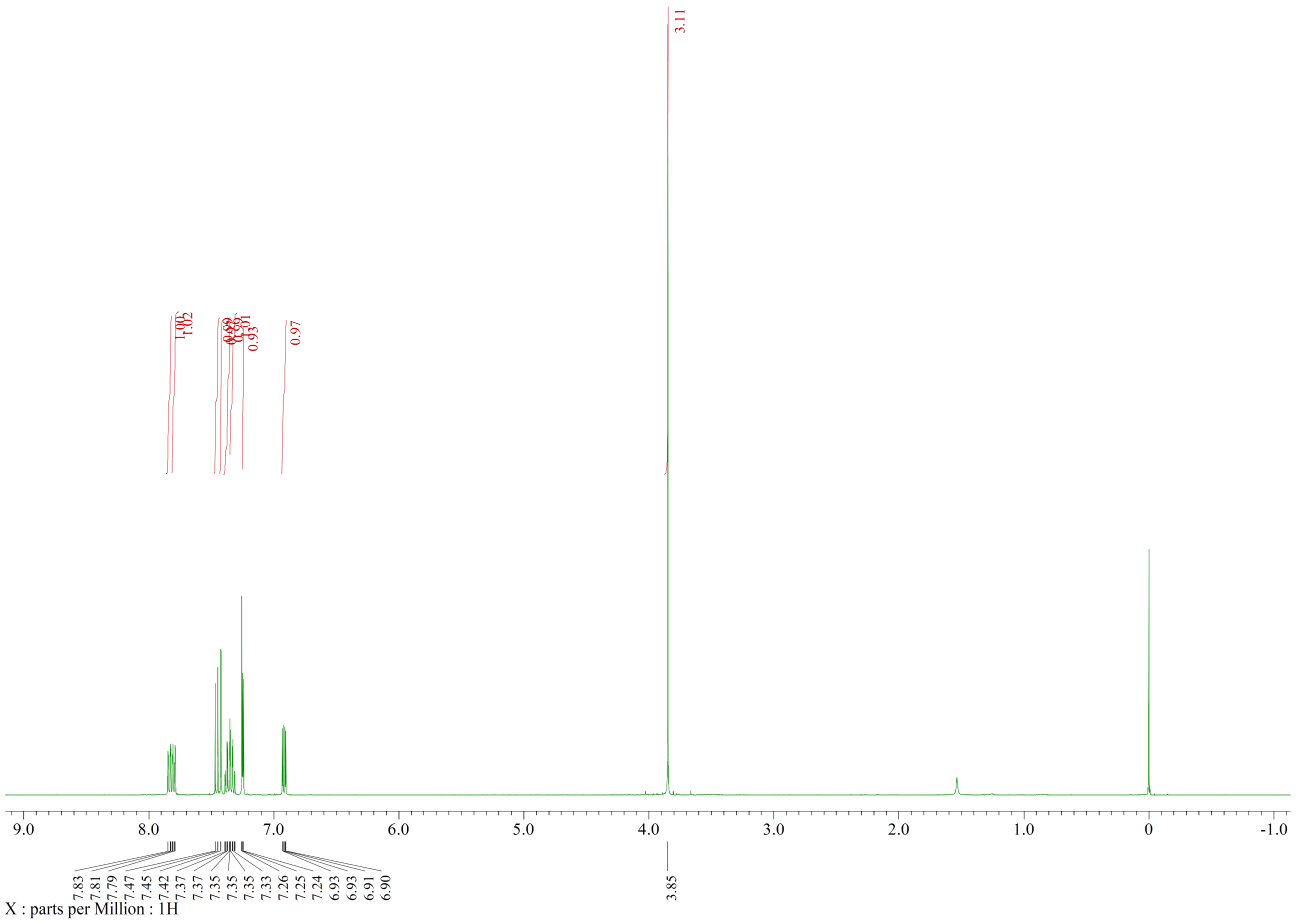

Figure S3. ^{1}H NMR spectrum of **7** (400 MHz, $CDCl_3$).

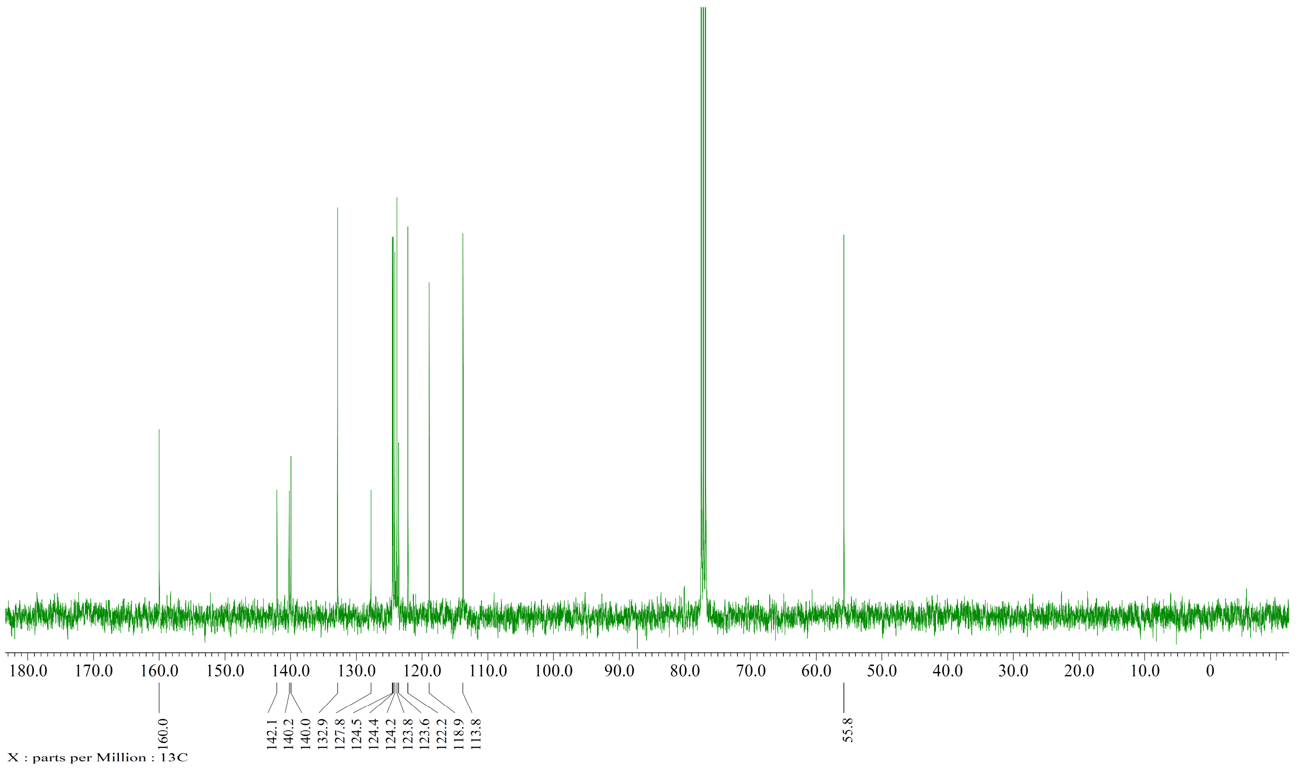

Figure S4. ^{13}C NMR spectrum of **7** (101 MHz, $CDCl_3$).

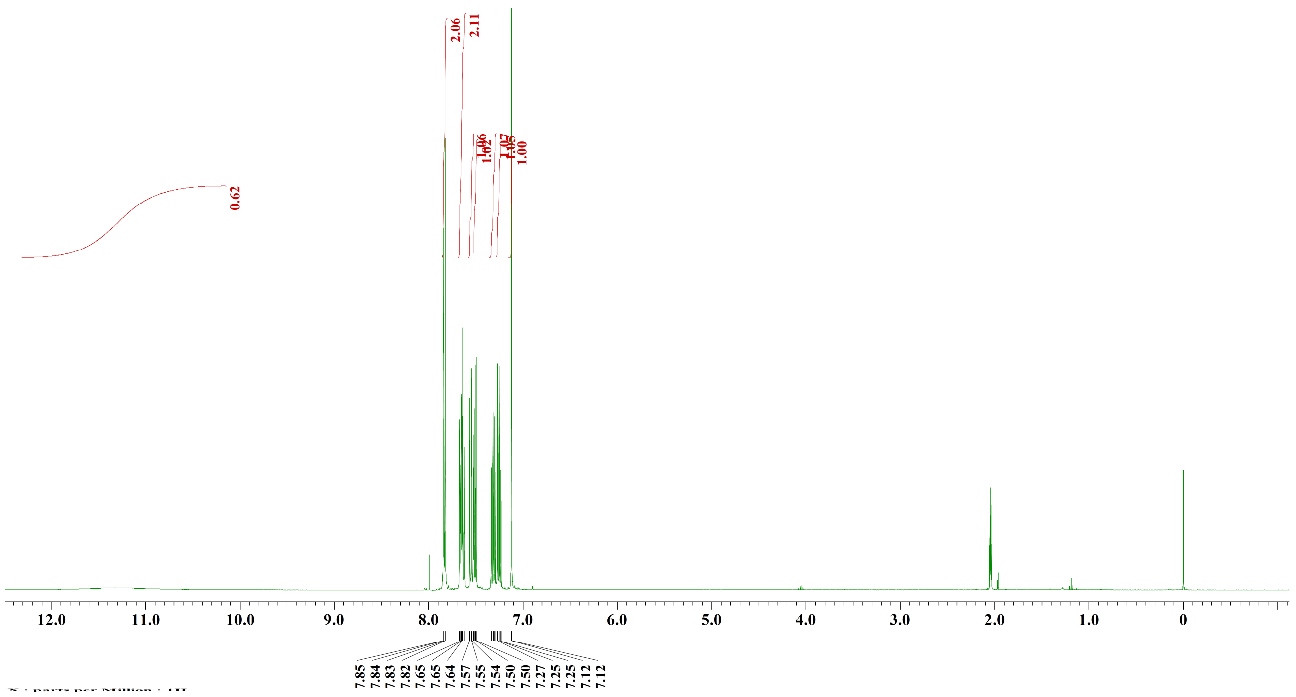

Figure S5. ^{1}H NMR spectrum of **13** (400 MHz, acetone-d_6).

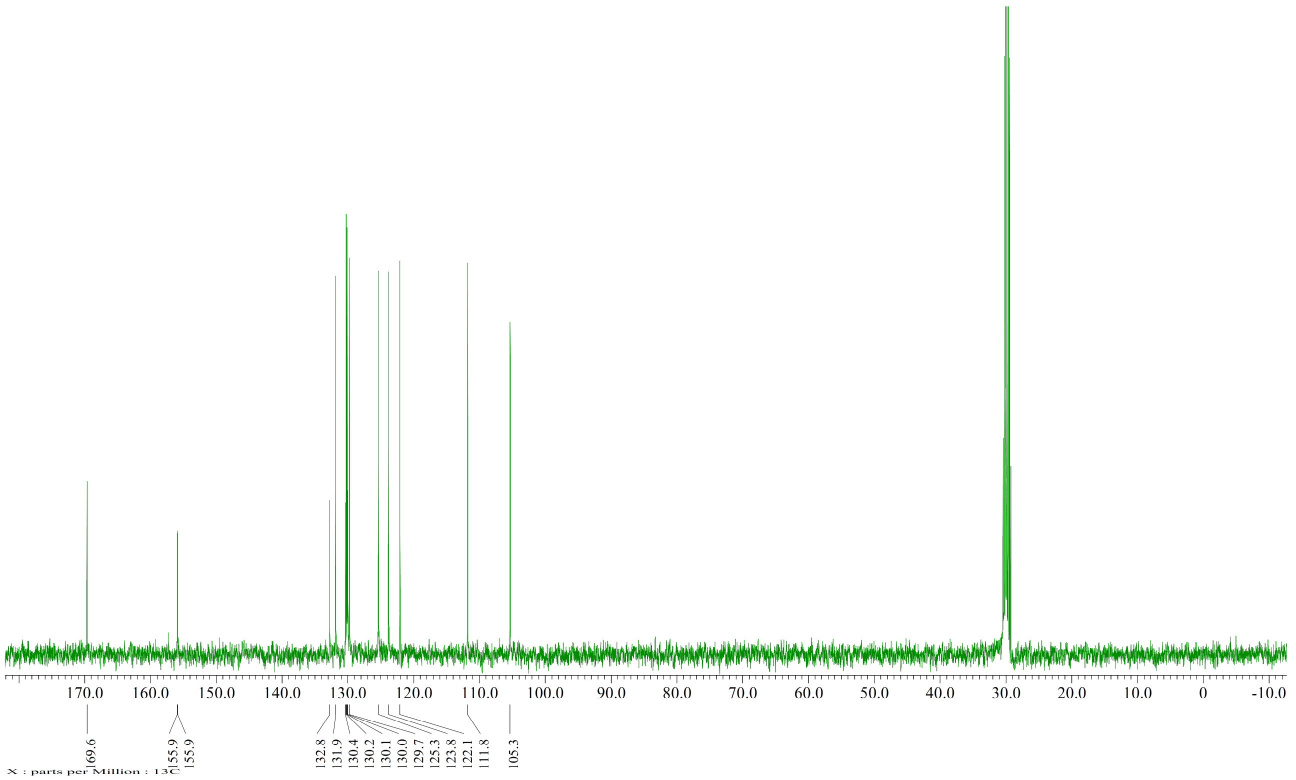

Figure S6. ^{13}C NMR spectrum of **13** (101 MHz, acetone-d_6).

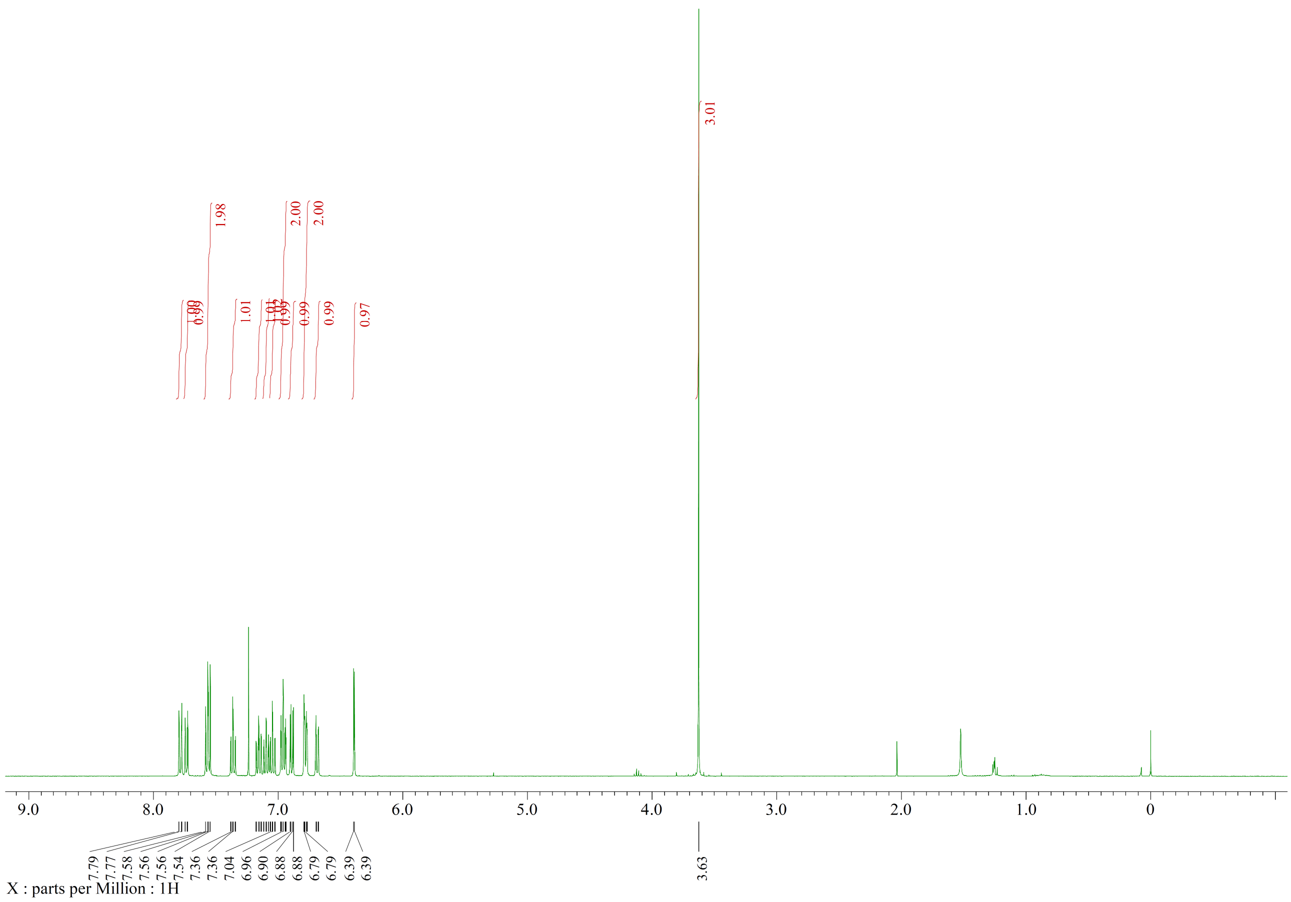

Figure S7. ^{1}H NMR spectrum of *rac*-**10** (400 MHz, $CDCl_3$).

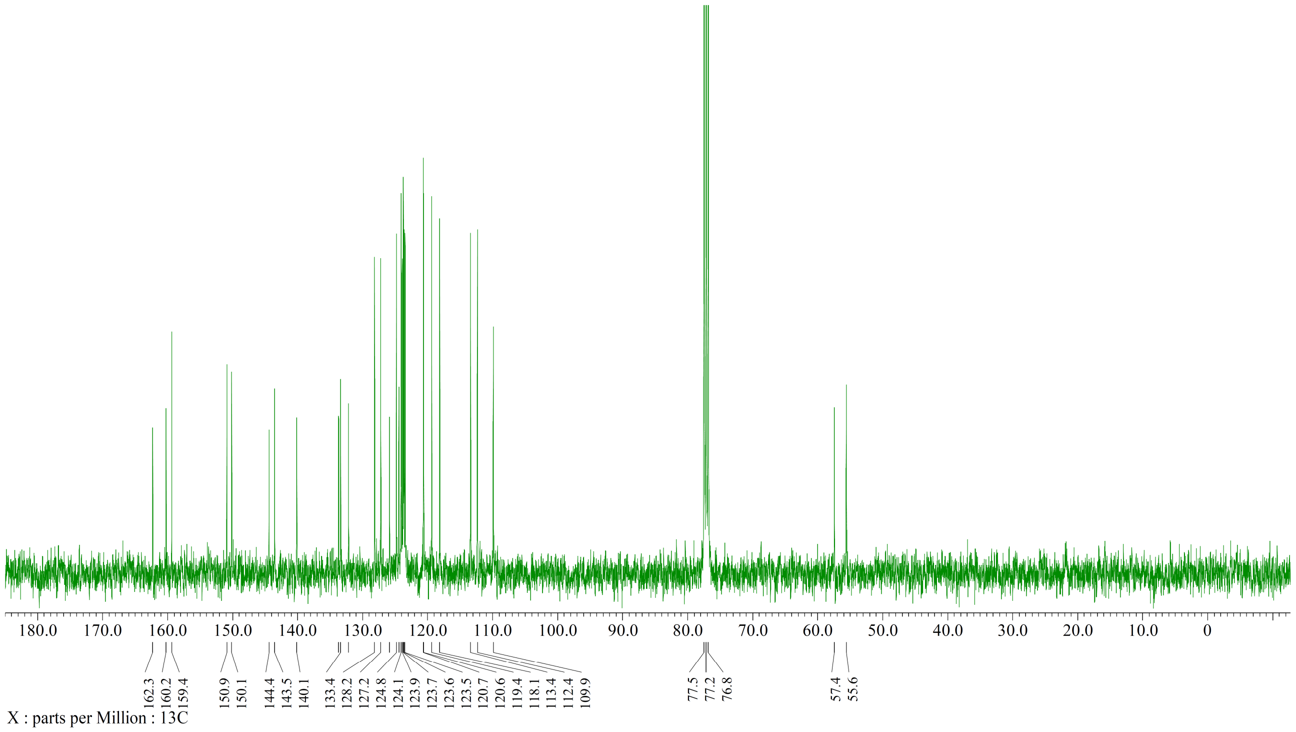

Figure S8. ^{13}C NMR spectrum of *rac*-**10** (101 MHz, $CDCl_3$).

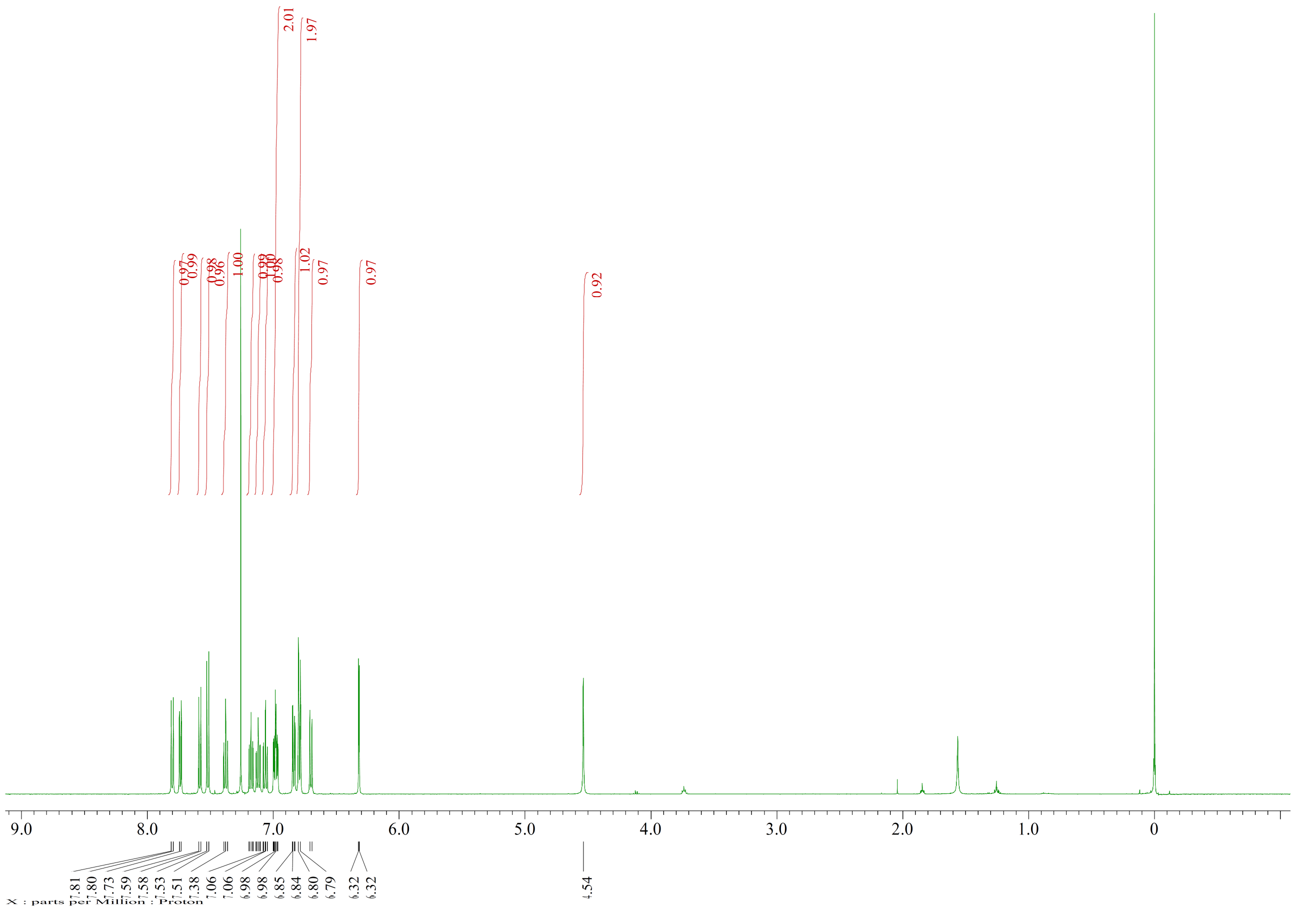

Figure S9. ^{1}H NMR spectrum of *rac*-**11** (500 MHz, $CDCl_3$).

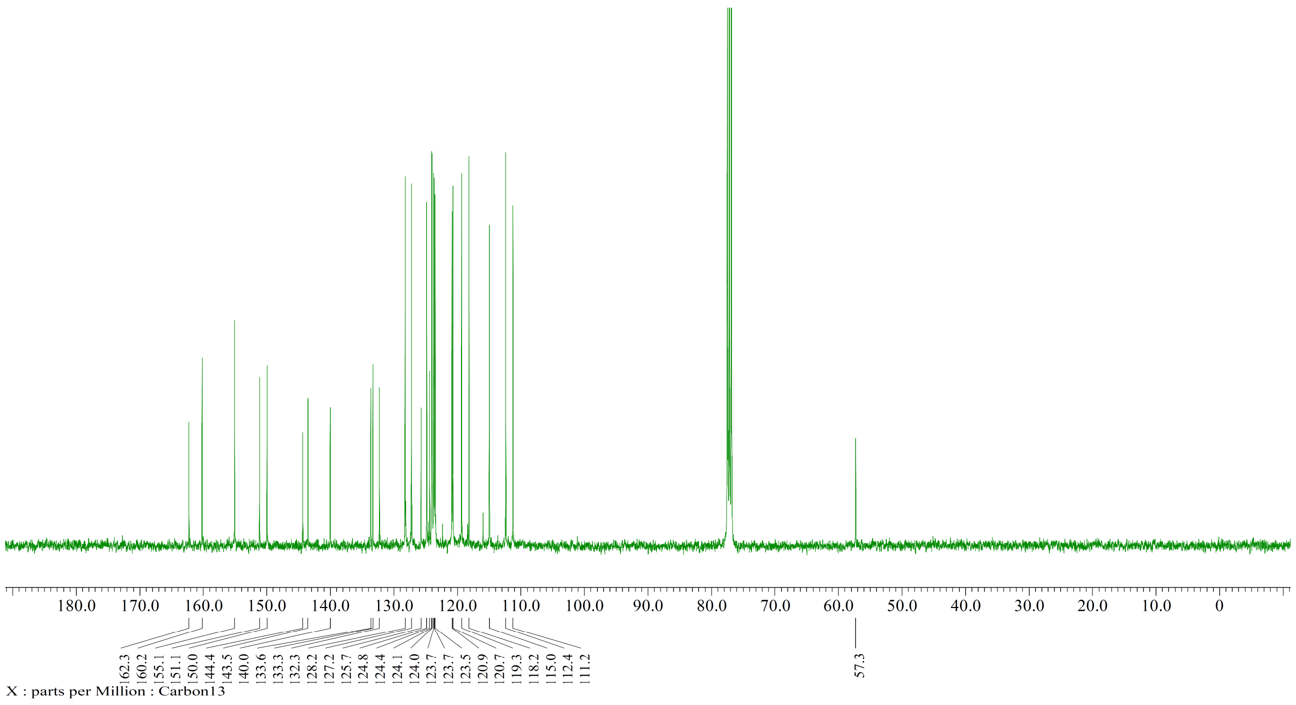

Figure S10. ^{13}C NMR spectrum of *rac*-**11** (101 MHz, $CDCl_3$).

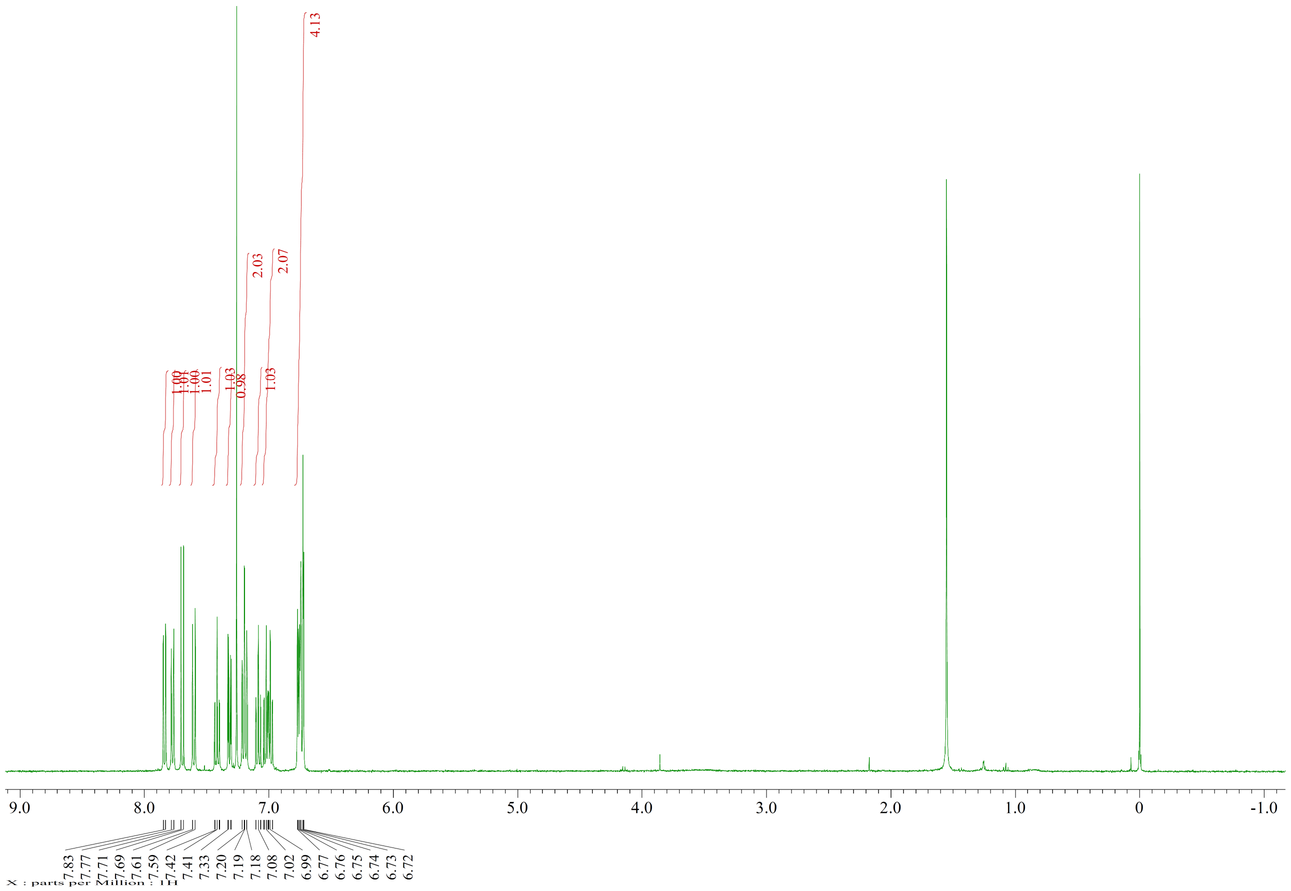

Figure S11. ^{1}H NMR spectrum of *rac*-**12** (400 MHz, $CDCl_3$).

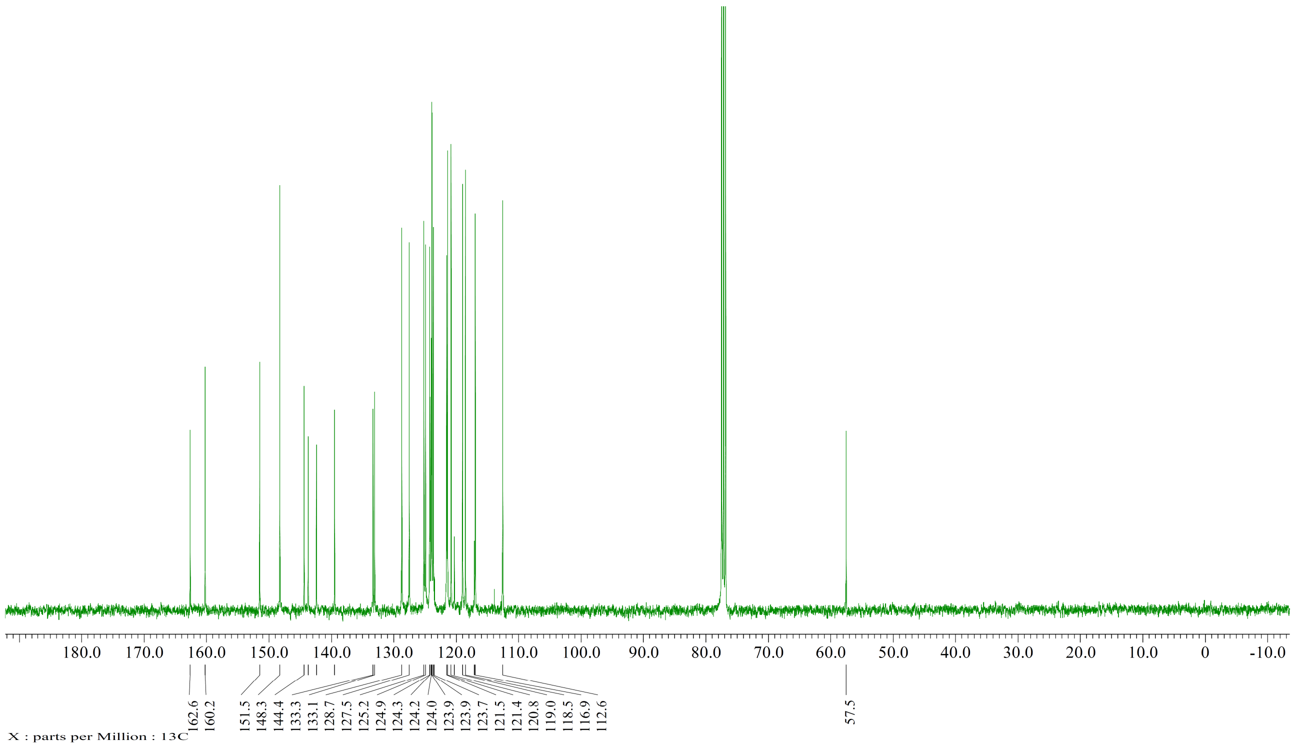

Figure S12. ^{13}C NMR spectrum of *rac*-**12** (101 MHz, $CDCl_3$).

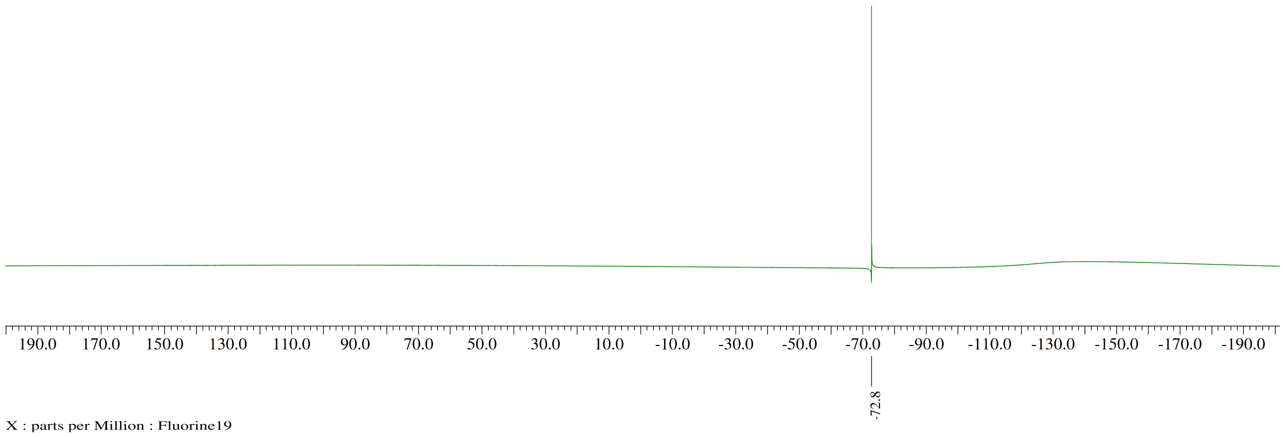

Figure S13. ^{19}F NMR spectrum of *rac*-**12** (376 MHz, $CDCl_3$).

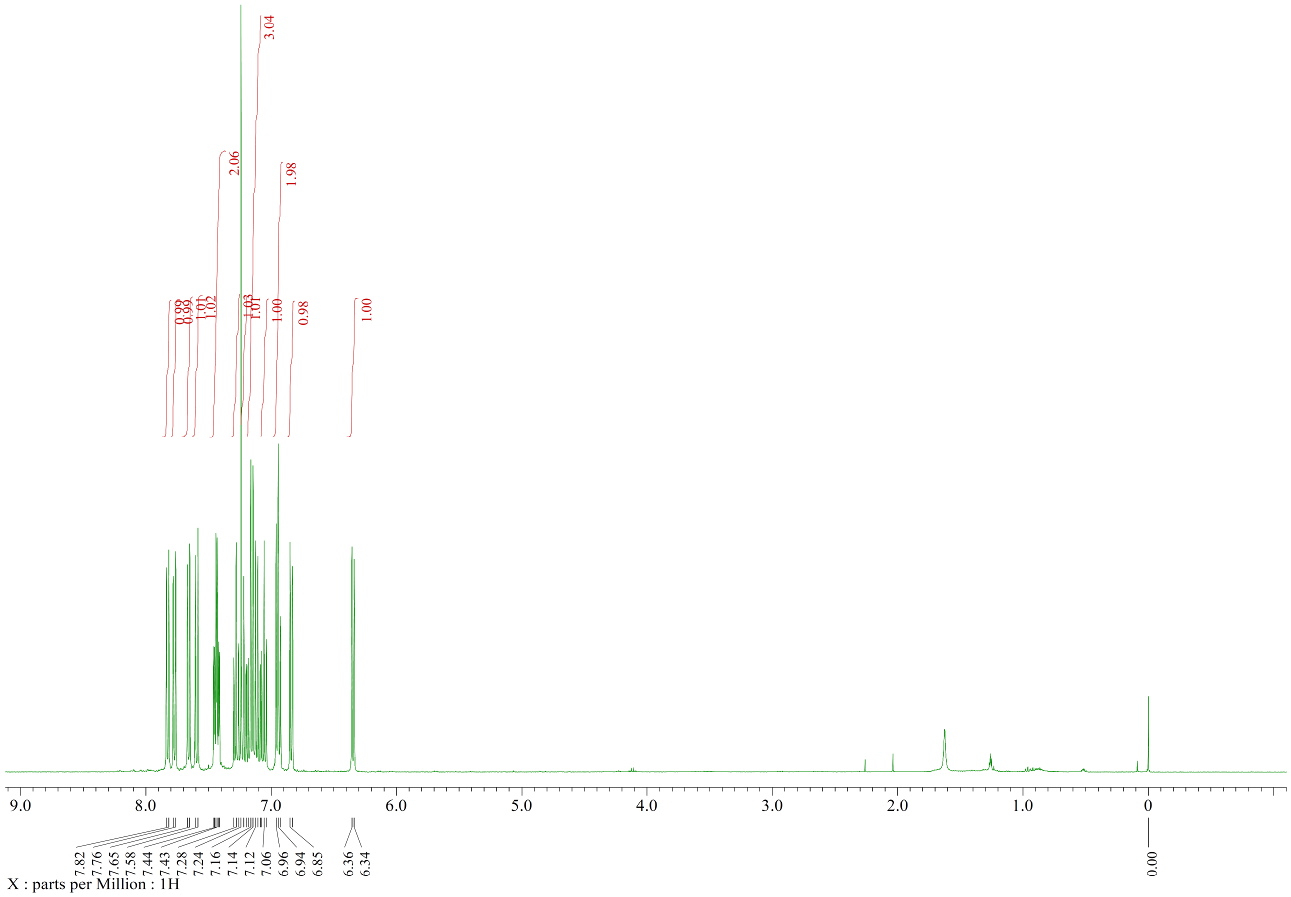

Figure S14. ^{1}H NMR spectrum of *rac*-**2** (400 MHz, $CDCl_3$).

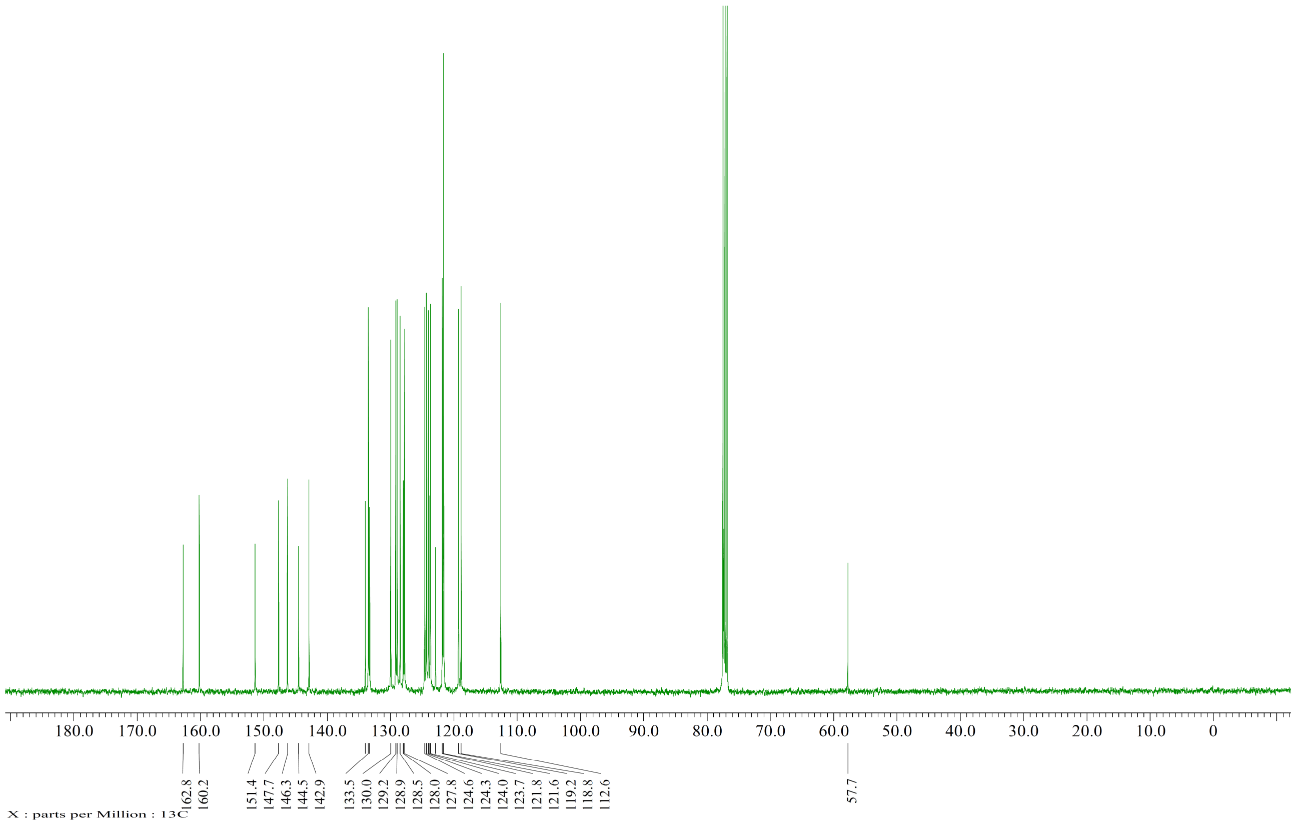

Figure S15. ^{13}C NMR spectrum of *rac*-**2** (101 MHz, $CDCl_3$).

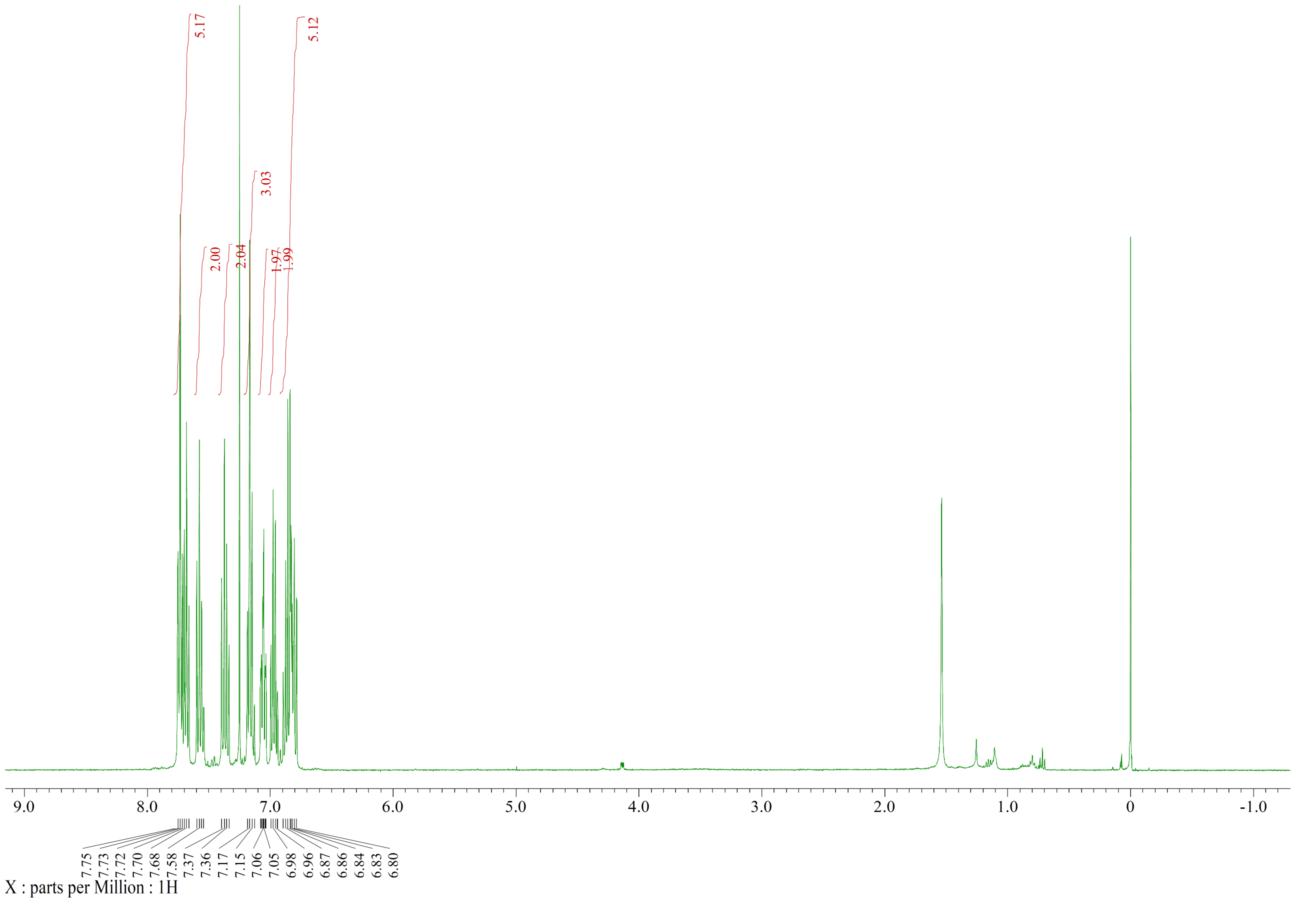

Figure S16. ^{1}H NMR spectrum of *rac*-**3** (400 MHz, $CDCl_3$).

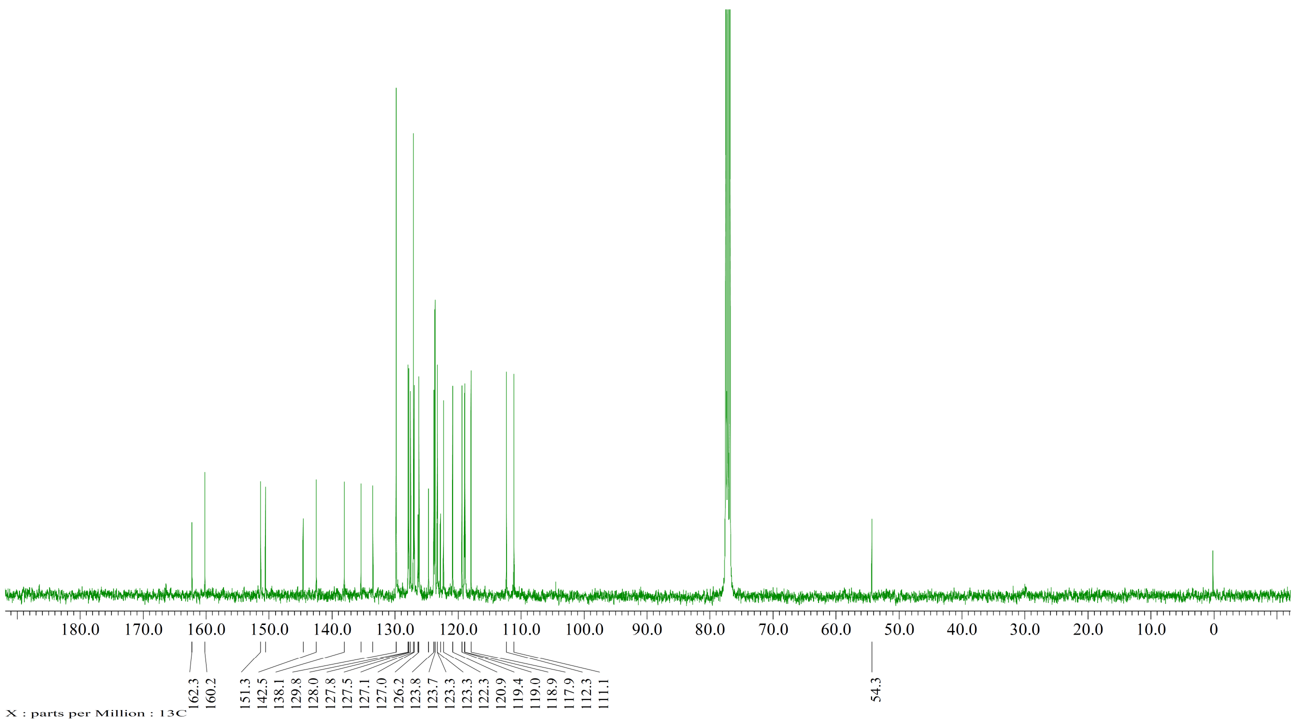

Figure S17. ^{13}C NMR spectrum of *rac*-**3** (101 MHz, $CDCl_3$).

Table S1. Crystallographic data and structure refinement details for *rac*-**1**

Formula	$C_{29}H_{16}OS$	
Formula weight	412.48	
Temperature	193(2) K	
Wavelength	1.54187 Å	
Crystal system	monoclinic	
Space group	$P2_1/n$	
Unit cell dimensions	a = 14.2753(3) Å	α = 90°
	b = 9.8401(2) Å	β = 114.4630(10)°
	c = 15.7643(3) Å	γ = 90°
Volume	2015.63(7) Å^3	
Z	4	
Density (calculated)	1.359 g/cm^3	
Absorption coefficient	1.566 mm^{-1}	
F(000)	856	
Crystal size	0.50 × 0.40 × 0.30 mm^3	
Theta range for data collection	3.519 to 68.226°	
Index ranges	–17<=h<=17, –11<=k<=11, –18<=l<=18	
Reflections collected	34993	
Independent reflections	3684 [R_{int} = 0.0448]	
Completeness to theta	100.0%	
Max. and min. transmission	0.625 and 0.492	
Refinement method	Full-matrix least-squares on F^2	
Data / restraints / parameters	3684 / 0 / 280	
Goodness-of-fit on F^2	1.046	
Final R indices [I>2sigma(I)]	R_1 = 0.0688, wR_2 = 0.1992	
R indices (all data)	R_1 = 0.0729, wR_2 = 0.2028	
Largest diff. peak and hole	1.219 and –0.486 e/Å^3	

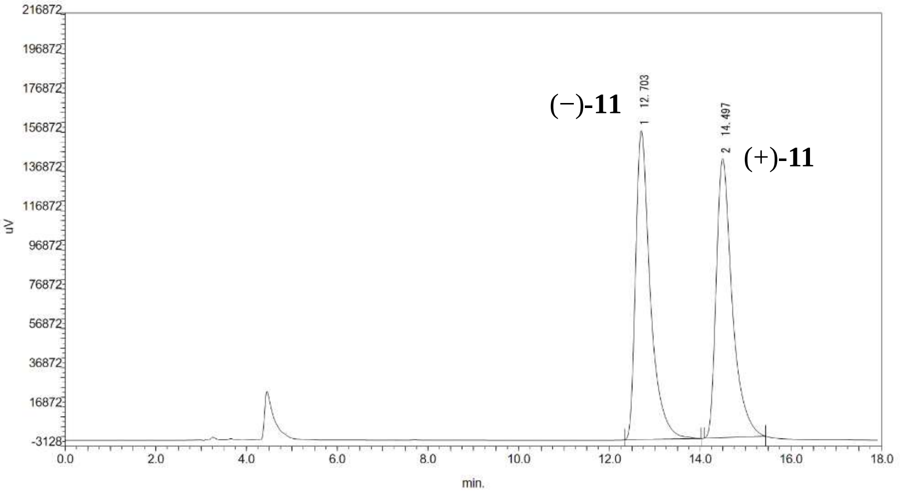

Figure S18. HPLC chart of *rac*-**11** with a DAICEL CHIEAPAK® IA-3 column (flow rate: 1.0 mL/min; eluent: hexane/$CHCl_3$ =50/50)].

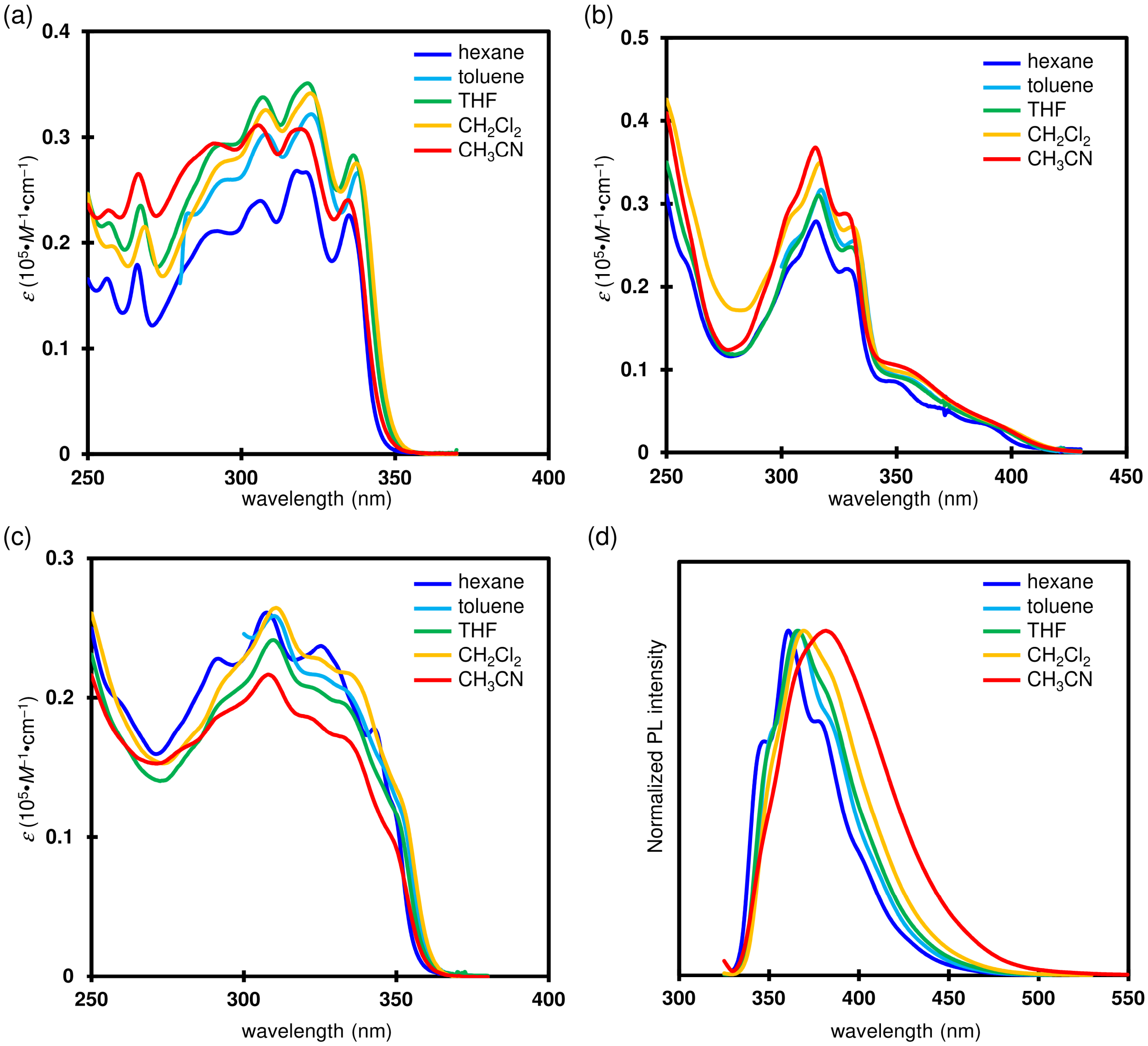

Figure S19. UV–vis absorption of (a) *rac*-**1**, (b) *rac*-**2**, and (c) *rac*-**3** and (d) PL spectra of *rac*-**1** in varioussolvents.

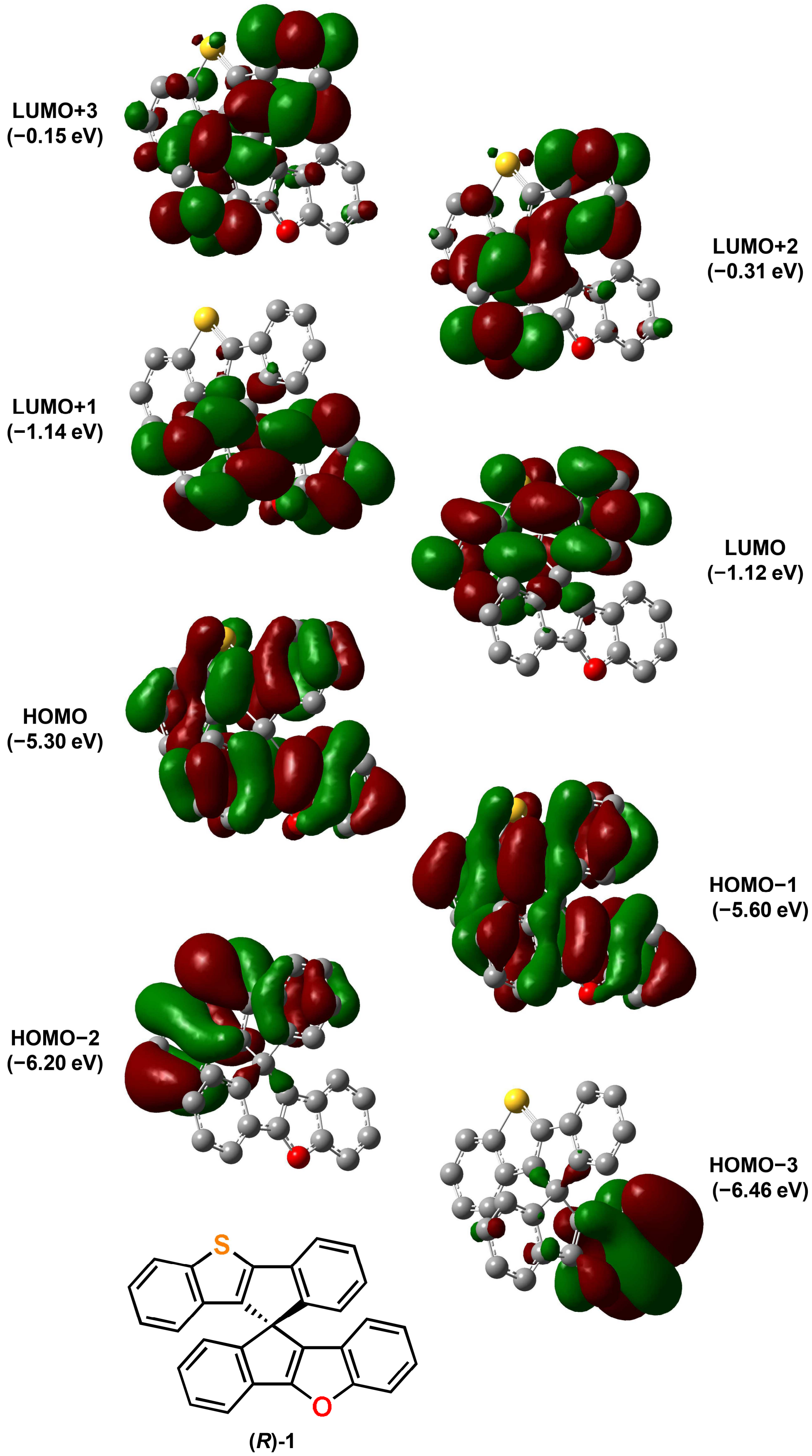

Figure S20. Molecular orbitals of (*R*)-**1** calculated by DFT method at the B3LYP/6-31G(d) level of theory.

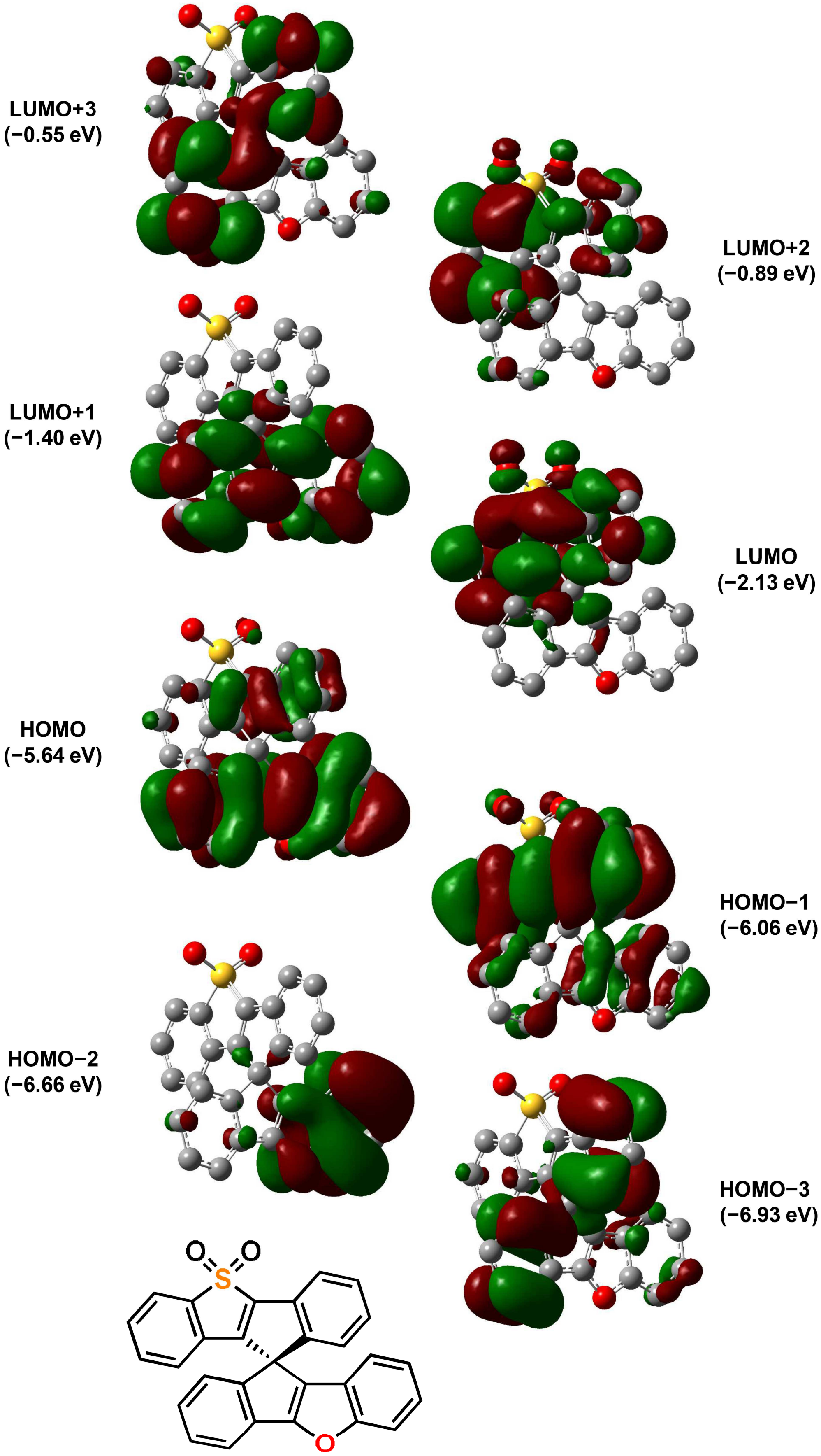

(*R*)-2

Figure S21. Molecular orbitals of (*R*)-**2** calculated by DFT method at the B3LYP/6-31G(d) level of theory.

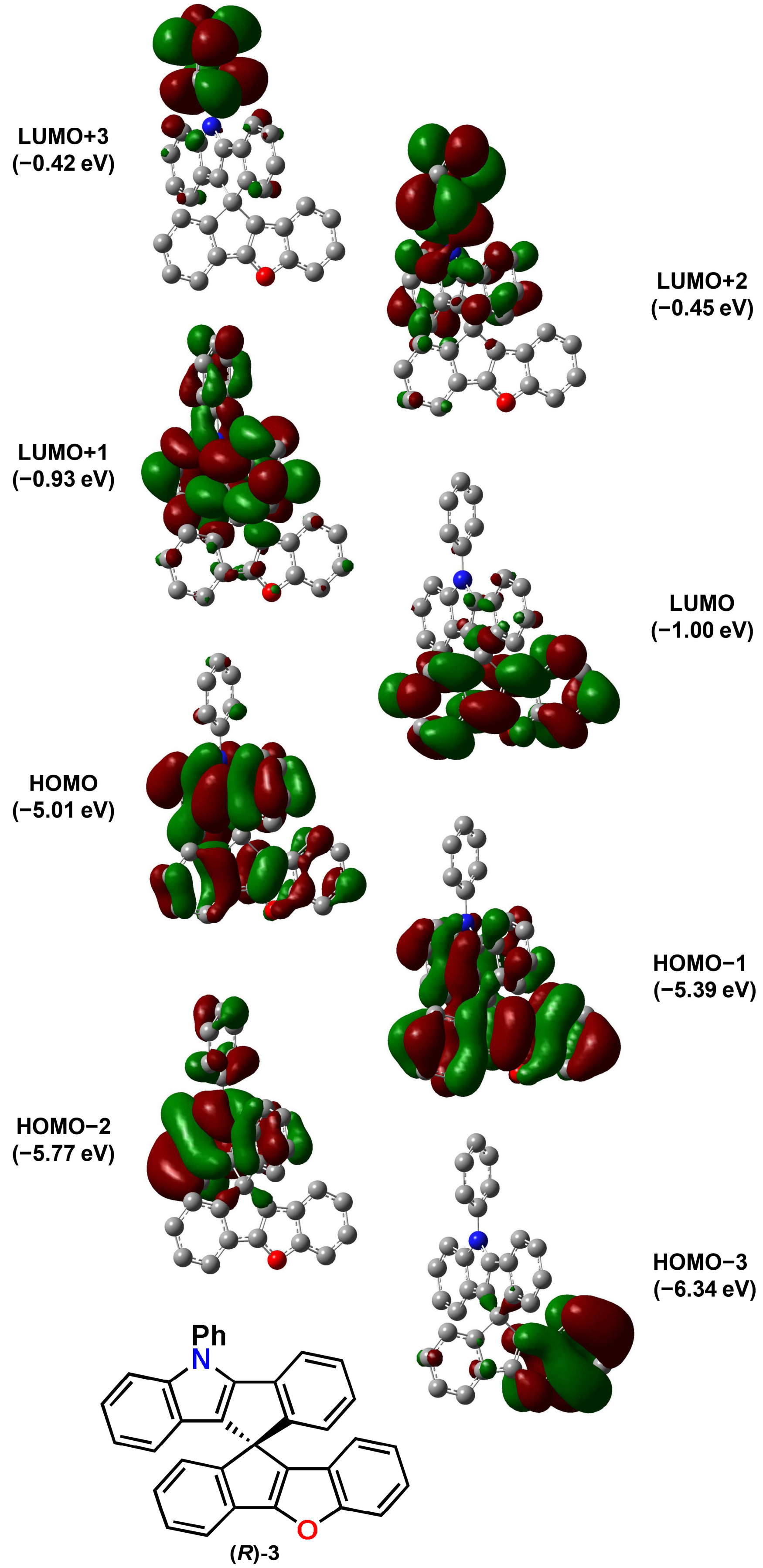

Figure S22. Molecular orbitals of (*R*)-**3** calculated by DFT method at the B3LYP/6-31G(d) level of theory.

Table S2. Coordinates (Å) and absolute energy of the optimized structure for (*R*)-**1**[a]

atom	x	y	z	atom	x	y	z
C	-1.952556	0.713568	-0.980512	C	0.908700	1.079226	4.474445
C	-1.218143	-0.066353	-0.130187	C	1.443432	0.319601	2.264482
C	-1.839531	-1.319636	0.184752	C	-0.632506	1.523600	2.642659
C	-3.090107	-1.441756	-0.483274	C	-0.286602	1.624666	3.998632
C	-1.274387	1.969390	-1.268473	C	1.789044	0.418749	3.611005
C	0.488915	4.113317	-1.427488	H	-1.563855	1.948774	2.278381
C	-1.608536	3.063616	-2.066023	H	-0.957772	2.132506	4.685612
C	-0.057493	1.947510	-0.547451	H	2.717980	-0.006735	3.978901
C	0.824751	3.013453	-0.625482	H	1.158335	1.167717	5.528240
C	-0.714295	4.136363	-2.138087	C	1.351249	-0.134100	-0.033818
H	-2.542224	3.083868	-2.621634	C	2.107253	-0.770625	-1.075829
H	1.761881	2.998158	-0.075379	C	4.119451	-2.159510	-2.436201
H	-0.958553	4.996642	-2.755308	C	3.267895	-1.257354	-0.419542
H	1.172467	4.954901	-1.497209	C	1.972016	-0.997547	-2.454161
C	-3.439973	-3.603759	0.486482	C	2.981736	-1.690256	-3.116919
H	-4.049561	-4.494158	0.612259	C	4.280988	-1.948368	-1.065557
C	-3.890638	-2.575737	-0.337352	H	1.097902	-0.639923	-2.989466
H	-4.843128	-2.655337	-0.853564	H	2.890242	-1.873593	-4.183872
C	-2.207916	-3.501219	1.155503	H	5.152311	-2.303533	-0.525078
H	-1.876364	-4.315491	1.793788	H	4.887809	-2.696505	-2.984852
C	-1.409765	-2.374312	1.011911	C	2.078038	-0.277245	1.107931
H	-0.459360	-2.299134	1.531127	O	3.248710	-0.951781	0.931479
C	0.077066	0.637554	0.252558	S	-3.460954	-0.017354	-1.471410
C	0.231493	0.872791	1.777457				

absolute energy E (B3LYP): −1588.14244225 au

[a]Calculated by DFT method [B3LYP/6-31G(d)]

Table S3. Coordinates (Å) and absolute energy of the optimized structure for (*R*)-**2**[a]

atom	x	y	z	atom	x	y	z
C	1.803199	0.692254	0.542259	C	-2.073923	0.318492	-2.070142
C	0.928803	-0.073780	-0.148048	C	-0.070306	1.476036	-2.817100
C	1.423834	-1.387385	-0.561583	C	-0.634024	1.549792	-4.099974
C	2.751328	-1.593741	-0.137523	C	-2.634676	0.391531	-3.343785
C	1.229610	1.973213	0.920810	H	0.913323	1.895287	-2.623287
C	-0.410213	4.171761	1.335144	H	-0.078539	2.029310	-4.900817
C	1.734381	3.063068	1.628716	H	-3.617652	-0.024736	-3.542759
C	-0.092537	1.979189	0.417426	H	-2.317822	1.082541	-5.357443
C	-0.915745	3.074830	0.623374	C	-1.610965	-0.082624	0.193679
C	0.897525	4.164603	1.829906	C	-2.193594	-0.676471	1.364610
H	2.749352	3.055500	2.014039	C	-3.976340	-1.988755	3.073718
H	-1.933554	3.085811	0.243045	C	-3.453977	-1.154790	0.921238
H	1.269115	5.025010	2.379146	C	-1.835666	-0.872148	2.707546
H	-1.044451	5.037346	1.504549	C	-2.734211	-1.527183	3.545282
C	2.794100	-3.763552	-1.124431	C	-4.358478	-1.808396	1.743200
H	3.315376	-4.689527	-1.348216	H	-0.879256	-0.521188	3.082666
C	3.450557	-2.755675	-0.401810	H	-2.471106	-1.686439	4.587108
H	4.473647	-2.884787	-0.061889	H	-5.312764	-2.158117	1.363305
C	1.478938	-3.581220	-1.555602	H	-4.652468	-2.495656	3.755959
H	0.983853	-4.370160	-2.114560	C	-2.517837	-0.238893	-0.809655
C	0.783654	-2.397645	-1.280618	O	-3.652699	-0.883206	-0.423648
H	-0.238017	-2.266189	-1.621193	S	3.365616	-0.148013	0.769709
C	-0.393850	0.662541	-0.319712	O	4.436725	0.521147	0.016621
C	-0.790564	0.861879	-1.805782	O	3.579763	-0.474882	2.187276
C	-1.898890	1.014691	-4.357308				

absolute energy E (B3LYP): -1738.49812445 au

[a]Calculated by DFT method [B3LYP/6-31G(d)]

Table S4. Coordinates (Å) and absolute energy of the optimized structure for (*R*)-**3**[a]

atom	x	y	z	atom	x	y	z
H	3.819280	-1.969443	-2.299164	H	2.837248	1.461118	2.427472
C	2.746894	-1.843217	-2.408289	H	-2.078842	2.310088	2.135841
C	-0.061901	-1.492334	-2.727400	H	1.695152	2.833057	4.148215
C	2.012611	-1.070374	-1.505228	H	-0.739722	3.263371	4.007949
C	2.057457	-2.442814	-3.458377	C	0.219568	-0.005684	-0.588616
C	0.667638	-2.273532	-3.613661	C	-1.003873	0.664490	0.000880
C	0.604643	-0.870800	-1.656896	C	-1.708381	1.659332	-0.958435
H	2.605235	-3.051408	-4.172580	C	-3.351858	3.205899	-2.598364
H	0.161649	-2.759000	-4.443657	C	-1.165936	2.741031	-1.632324
H	-1.131470	-1.355773	-2.856841	C	-3.083343	1.346524	-1.109227
N	2.452128	-0.352827	-0.380998	C	-3.911316	2.114256	-1.926097
C	3.799214	-0.247609	0.064449	C	-1.996817	3.516070	-2.455301
C	6.450753	-0.040720	0.930474	H	-0.112042	2.984358	-1.527340
C	4.526897	-1.402527	0.375331	H	-4.963337	1.869052	-2.038098
C	4.401514	1.010632	0.184376	H	-1.579928	4.365937	-2.988695
C	5.720458	1.110244	0.627301	H	-3.979056	3.817758	-3.241034
C	5.851665	-1.294961	0.798554	C	-2.166515	-0.247885	0.344053
H	4.047052	-2.372857	0.295496	C	-2.540320	-1.407670	1.104213
H	3.835101	1.899316	-0.076479	C	-4.042273	-3.485300	2.227288
H	6.181280	2.089724	0.721109	C	-3.924395	-1.573064	0.835548
H	6.411453	-2.194702	1.038615	C	-1.911891	-2.321577	1.964148
H	7.480450	0.039393	1.267115	C	-2.672345	-3.349764	2.514993
C	1.341736	0.286365	0.148832	C	-4.694258	-2.591056	1.375887
C	1.020925	1.121928	1.300195	H	-0.854925	-2.226129	2.192645
C	-0.251858	2.662355	3.245562	H	-2.198455	-4.064527	3.182170
C	1.771085	1.647946	2.354079	H	-5.750322	-2.680813	1.143025
C	-0.375279	1.362514	1.228196	H	-4.605273	-4.299646	2.674185
C	-1.008687	2.129165	2.192377	C	-3.295528	0.173445	-0.287777
C	1.120858	2.419827	3.323259	O	-4.391034	-0.594486	-0.025874

absolute energy E (B3LYP): −1476.35246608 au

[a]Calculated by DFT method [B3LYP/6-31G(d)]

Table S5. The Selected Absorption of (*P*)-**1–3** Calculated by TD–DFT Method at the B3LYP/6-31G(d) Level of Theory

	excited state	transition energy (eV)	wavelength (nm)	main transition configuration (CI expansion coefficient)	oscillator strength f	Rotatory Strength (10^{-40} erg·esu·cm/gauss)		transition electric dipole moments (a.u.)			transition magnetic dipole moments (a.u.)		
						$R_{velocity}$	R_{length}	x	y	z	x	y	z
(*R*)-**1**	1	3.5246	352	HOMO → LUMO (0.66852)	0.0402	−28.0408	−27.9770	0.1674	0.6420	−0.1595	−0.2803	0.4060	0.5962
	2	3.6405	341	HOMO → LUMO+1 (0.64566)	0.0702	−0.0310	1.1379	0.3880	−0.3638	−0.7103	−0.3981	−0.8916	0.2459
	3	3.9846	311	HOMO−1 → LUMO (0.65536)	0.3077	44.7448	46.0587	−0.4951	−1.5800	0.6405	0.4860	−0.5350	−1.2491
	4	4.0486	306	HOMO−1 →LUMO+1 (0.62751)	0.3357	−48.7745	−47.2841	0.7241	−0.7894	−1.4956	−0.5213	−1.5154	0.4134
	5	4.3139	287	HOMO−2 → LUMO (0.46858)<br>HOMO → LUMO+2 (0.42326)	0.0621	−29.3488	−30.6337	−0.3486	−0.2343	−0.6412	0.1461	−0.2047	−0.2073
	6	4.3596	284	HOMO → LUMO+2 (0.47078)<br>HOMO−2 → LUMO (−0.36831)	0.0303	52.5987	53.2310	0.6404	0.2591	−0.7063	0.0016	−0.1231	0.2761
(*R*)-**2**	1	2.8963	428	HOMO → LUMO (0.70392)	0.0110	−9.3543	−8.9640	0.0463	0.3637	0.1423	0.0297	0.1548	−0.1380
	2	3.4462	360	HOMO−1 → LUMO (0.69430)	0.2207	−4.7145	−2.1271	−0.4761	1.4650	0.4905	−0.1184	0.0185	−0.1517
	3	3.8275	324	HOMO → LUMO+1 (0.64303)	0.2119	1.1459	3.2585	−0.4315	−0.5325	1.3377	0.4585	−1.5043	−0.4612
	4	4.0079	309	HOMO−2 → LUMO (0.70213)	0.0003	0.3028	0.3081	−0.0007	0.0348	0.0458	−0.0187	−0.0589	0.0160
	5	4.1177	301	HOMO−1 → LUMO+1 (0.61428)	0.1646	42.0854	46.3874	0.3768	0.3608	−1.1662	−0.2803	1.1407	0.4311
	6	4.1398	300	HOMO−3 → LUMO (0.65245)	0.0098	8.4063	8.7941	0.2188	0.2190	0.0334	−0.2234	−0.0112	0.4204
(*R*)-**3**	1	3.4366	361	HOMO → LUMO (0.68501)	0.0330	−31.5369	−31.2033	0.1402	0.5524	−0.2589	−0.1432	0.6092	0.7108
	2	3.6098	343	HOMO → LUMO+1 (0.65694)	0.1078	−1.1846	−0.7116	−0.1112	−0.5955	−0.9227	−0.0758	−0.7913	0.5165
	3	3.9377	315	HOMO → LUMO+2 (0.47770)<br>HOMO−1 → LUMO+1 (0.44947)	0.0767	3.6022	4.9026	−0.5520	−0.3286	−0.6180	0.1269	−0.1278	−0.0117
	4	3.9791	312	HOMO → LUMO+2 (0.46482)<br>HOMO−1 → LUMO+1 (−0.34814)<br>HOMO−1 → LUMO (0.24808)	0.0882	166.7527	168.7944	−0.3028	0.0875	0.8975	0.1065	0.3798	−0.7989
	5	4.0281	308	HOMO → LUMO+3 (0.53651)<br>HOMO−1 → LUMO (−0.40656)	0.1286	192.2917	198.3647	0.1567	0.6460	−0.9283	−0.1526	0.6956	1.3648
	6	4.0481	306	HOMO−1 → LUMO (0.44502)<br>HOMO → LUMO+3 (0.36395)<br>HOMO−1 → LUMO+1 (0.32045)	0.2607	−376.3605	−387.3010	−0.0165	−1.5624	0.4331	0.0647	−1.3542	−1.0889
